# Screening and identification of two novel phosphate-solubilizing *Pyrenochaetopsis tabarestanensis* strains and their role in enhancing phosphorus uptake in rice

**DOI:** 10.3389/fmicb.2024.1494859

**Published:** 2025-01-09

**Authors:** Xiaozhe Bao, Haifei Lu, Jinyao Zhao, Taotao Yang, Longmei Wu, Jixiang Zou, Qingchun Chen, Bin Zhang

**Affiliations:** ^1^Rice Research Institute, Guangdong Academy of Agricultural Sciences/Guangdong Key Laboratory of New Technology in Rice Breeding/Guangdong Rice Engineering Laboratory/Key Laboratory of Genetics and Breeding of High Quality Rice in Southern China (Co-construction by Ministry and Province), Ministry of Agriculture and Rural Affairs, Guangzhou, China; ^2^Nanjing Institute of Environmental Science, Ministry of Ecology and Environment of the People's Republic of China, Nanjing, China; ^3^College of Agriculture and Biology, Zhongkai University of Agriculture and Engineering, Guangzhou, China

**Keywords:** antifungal activity, auxins and phytohormones, biofertilization, organic acid secretion, phosphate-solubilizing fungi (PSF), plant phosphorus absorption

## Abstract

Low phosphorus (P) use efficiency significantly impacts rice yields. An environmentally friendly approach to increase phosphorus absorption and utilization in rice involves the exploration of phosphorus-solubilizing fungal resources. This study aimed to isolate and characterize fungal strains from the rice rhizosphere and assess their phosphate solubilization capabilities, plant-growth-promoting (PGP) traits, and mechanisms involved. An initial comparative sequence analysis of the hypervariable regions of the ITS rDNA and morphological analysis identified two strains belonging to the genus *Pyrenochaetopsis*, designated *Pyrenochaetopsis tabarestanensis* WFY-1 (PtWFY-1) and WFY-2 (PtWFY-2). Both strains demonstrated the ability to solubilize tricalcium phosphate, magnesium phosphate, phosphate rock powder, and calcium phytate phosphorus *in vitro* through acidification via the exudation of oxoglutaric acid, acetic acid, citric acid, and pyruvic acid. The amounts of oxoglutaric acid, acetic acid, citric acid, and pyruvic acid secreted were 1,900.03, 1,478.47, 579.11, and 685.90 mg L^−1^, respectively, for the PtWFY-1 strain and 2,441.67, 1,519.18, 867.65, and 888.30 mg L^−1^, respectively, for the PtWFY-2 strain relative to the control (0.00 mg L^−1^). These organic acids acidify the rhizosphere, increasing the availability of phosphorus for plant uptake. Inoculation with PtWFY-1 increased available soil P by 5.8% after 30 days, increasing the plant P concentration by 69.8% and the dry weight of the rice seedlings by 24.5%. Similarly, the PtWFY-2 strain increased these parameters by 7.7%, 60.3%, and 14.5%, respectively. PtWFY-1 showed slightly stronger effects on P availability and plant growth compared to PtWFY-2. The secretion of phytohormones was responsible for the growth promotion in rice by the PtWFY-1 and PtWFY-2 strains, along with P absorption The principal phytohormone in the PtWFY-1 and PtWFY-2 broths was L-tryptophan, which is a precursor substance for IAA synthesis, accounting for 84.68% and 83.46%, respectively. Assessment of the antifungal activities of the PtWFY-1 and PtWFY-2 strains against *Magnaporthe oryzae* demonstrated that rice grew healthier, indirectly promoting rice phosphorus absorption. These findings highlight the potential of using *Pyrenochaetopsis* strains as biofertilizers to sustainably improve phosphorus use efficiency in rice agriculture.

## 1 Introduction

Rice (*Oryza sativa* L.) is a crucial crop worldwide, with demand increasing alongside the growing world population (Tiwari et al., 2017). Phosphorus (P) is essential for rice growth, yet the majority of phosphorus fertilizer applied to the soil combines with ions such as Ca^2+^, Fe^2+^, Fe^3+^, and Al^3+^ to form insoluble phosphorus, rendering the phosphate fertilizer utilization rate during the rice growing season no more than 30% (Bünemann, 2015; Dash and Dangar, 2017). Additionally, the use of phosphate fertilizer poses challenges, including high costs, significant environmental pollution, and short-term effects. Therefore, developing and utilizing microbial fertilizers to improve phosphorus use efficiency in rice is urgently needed.

Phosphate-solubilizing microorganisms (PSMs) are capable of converting insoluble phosphate into effective phosphorus in the soil. Compared with phosphate-solubilizing bacteria (PSB), phosphate-solubilizing fungi (PSF) generally exhibit greater solubilization ability and more active metabolic capacity (Antarikanonda et al., 1990; Kucey, 1983), and their genetic traits are considerably more stable (Kaul et al., 2019). However, fewer phosphorus-solubilizing fungal species have been isolated than phosphorus-solubilizing bacteria, and many phosphate-solubilizing fungal resources remain unexplored. To date, several phosphate-solubilizing fungal species, including *Aspergillus, Penicillium, Trichoderma, Rhizopus, Sclerotium, Talaromyces*, and *Fusarium*, have been isolated from plants and their root rhizospheres (Alori et al., 2017; Kalayu, 2019). These PSFs have also been isolated from rice plants and paddy soils. For example, Deepa et al. (2010) isolated 12 strains of fungi with efficient phosphate solubilizing ability from paddy soils belonging to the genera *Alternaria, Cladosporium, Aspergillus, Penicillium, Fusarium*, and *Rhizoctonia*. Mwajita et al. (2013) reported a total of 21 fungal strains with phosphate solubilization functions in the rice leaf phyllosphere, root rhizosphere, and soil, including *Penicillium, Aspergillus, Fusarium*, and *Xylaria*. To date, few species of phosphate-solubilizing fungi have been isolated, and many phosphate-solubilizing fungal resources remain undiscovered in paddy fields. Therefore, the isolation of phosphate-solubilizing fungi in this study will supplement the resources of phosphate-solubilizing fungi in rice fields and deepen our understanding of their diversity and functions.

Recent studies have shown that PSF's main mechanisms include rhizosphere acidification (producing organic acids and inorganic acids to reduce the soil pH and dissolve phosphate) (Yadav et al., 2015), chelation (generating chelates to bind metal ions and release phosphate ions) (Paul and Sinha, 2013; Sarker et al., 2014), and mineralization (producing enzymes to convert insoluble phosphorus into plant-absorbable forms) (Raliya et al., 2016). Overall, the primary mechanisms of phosphorus-solubilizing fungi involve acidification, chelation, and exchange reactions associated with the exudation of low-molecular-weight organic acids. The promotion of plant growth and disease resistance also indirectly increases the absorption and utilization of phosphorus by plants (Naeimi et al., 2010; Chagas et al., 2017).

*Pyrenochaetopsis* Gruyter, Aveskamp and Verkley, gen. nov, established by de Gruyter et al. (2010), is a genus belonging to the family Pyrenochaetopsidaceae, order Pleosporales, class Dothideomycetes in the Ascomycetes (de Gruyter et al., 2010; Valenzuela-Lopez et al., 2018). It is widely distributed and comprises the most common type of microorganisms in paddy ecosystems, accounting for about 4.0%−6.6% of the microbial population (Papizadeh et al., 2017; Chen et al., 2020). Previous studies have highlighted multiple biological functions of *Pyrenochaetopsis* spp. For example, de Gruyter et al. (2010) reported their direct involvement in regulating soil respiration and enzyme activities in the soil carbon cycle and their strong capacity for solubilizing insoluble soil carbon (organic matter, etc.). Bai et al. (2019) noted that *Pyrenochaetopsis* spp. were found to significantly affect soil CO_2_ emissions. Furthermore, Xun et al. (2020) reported a positive correlation between the relative abundance of *Pyrenochaetopsis* spp. in paddy soil and N_2_O release. Additionally, factors such as soil organic carbon levels, Zn concentrations, and biochar addition were shown to influence the distribution and ecological functions of *Pyrenochaetopsis* spp. in paddy fields (Zheng et al., 2016; Chen et al., 2020). To date, there have been no reports on *Pyrenochaetopsis* spp. with phosphate-solubilizing functions and promoting effects on phosphorus absorption in rice.

This study is the first to isolate *Pyrenochaetopsis* strains with phosphate-solubilizing capabilities from the rice rhizosphere. The objectives were (i) to evaluate the efficiency and mechanisms of these two fungal strains in dissolving insoluble phosphate and (ii) to assess their ability to improve P availability in soils, increase rice P uptake, and elucidate the mechanisms involved. This research is part of ongoing efforts to identify potential PSM inoculants as novel alternatives for phosphorus management in agricultural soils. This research further elucidates the phosphate solubilization mechanisms of phosphate-solubilizing fungi in paddy fields and provides theoretical support for the exploration and utilization of functional phosphate-solubilizing fungi in paddy fields.

## 2 Materials and methods

### 2.1 Locations and collection of rice root samples

The study was conducted at the Dafeng experimental base (113°22′E, 23°09′N) of the Guangdong Academy of Agricultural Sciences, Guangdong Province, southern China. The area is characterized by a subtropical monsoon climate with an average annual air temperature of 21.8°C and an average annual precipitation of 1,694 mm. Roots of the Wufengyou615 variety were collected in October 2019 during the heading stage of the rice plant.

To ensure the reliability of the results, five healthy rice plants at the tillering stage were randomly sampled from different locations, placed on ice in a cooler box, and promptly transported to the laboratory. The samples were refrigerated at 4°C for later use and processed within 24 h of collection.

### 2.2 Isolation of *Pyrenochaetopsis tabarestanensis* strains from the rice rhizosphere

One gram soil tightly adhering to the roots of five rice plants was separately dissolved in 100 mL of sterile deionized water in 250 mL Erlenmeyer flasks. The flasks were incubated on a thermostatic shaker for 30 min at 28°C and 180 rpm. The supernatant was serially diluted to concentrations ranging from 10^−1^ to 10^−7^ with sterile deionized water and plated on 10 cm petri dishes containing potato dextrose agar (PDA) medium: 200 g of potato was cut into small pieces, boiled for 30 min with 1,000 mL of water, and then filtered. The filtrate was mixed with 20 g of glucose and 20 g of agar, and deionized water was added to 1,000 mL. The medium was sterilized at 121°C for 20 min, cooled, and stored for later use. The fungal discs were incubated at 28 ± 2°C for 5–7 days in an incubator. Fungal colonies were purified by repeatedly transferring a single hyphal tip to PDA agar medium.

### 2.3 Identification of *Pyrenochaetopsis tabarestanensis* strains

The fungal isolates were identified on the basis of their morphological characteristics and genetic analysis. The pure isolates of *Pyrenochaetopsis tabarestanensis* obtained from the above isolation and purification process were transferred to oatmeal medium for fungal morphology identification. The universal ITS rRNA sequence of the fungal isolates was used for genetic identification. The primers used for PCR amplification were ITS1F (5′-CTTGGTCATTTAGAGGAAGTAA-3′) and ITS2R (5′-GCTGCGTTCTTCATCGATGC-3′) (Adams et al., 2013). DNA extraction of the isolates was conducted following the procedure specified by the manufacturers of the Fungi kit (Omega Bio-Tek, Inc., US). DNA quality was determined with a NanoDrop 2000 spectrophotometer (Thermo Scientific, USA). PCR amplification of the target sequence was performed as previously described (Qarni et al., 2021). Amplified fragments were checked and purified by using QIA QuickPCR purification kit (QIAGEN) and then sequenced at BGI Genomics Co., Ltd. (Shenzhen, China).

The nucleotide sequences so generated were compared using National Centre of Biotechnology Information (NCBI) BLAST method (https://blast.ncbi.nlm.nih.gov/Blast.cgi), and the ITS sequence showed 99.77% homology to *Pyrenochaetopsis tabarestanensis*. The sequences of the novel isolates were deposited in the NCBI GenBank database under the accession numbers PP658459 and PP658460. The newly identified fungal strains were named *Pyrenochaetopsis tabarestanensis* WFY-1 (PtWFY-1) and WFY-2 (PtWFY-2), and they were preserved at the Guangdong Microbial Culture Collection Center (GDMCC), Guangzhou, China, accession numbers GDMCC No. 61861 and GDMCC No. 61862. The phylogenetic tree was constructed by the neighbor-joining (NJ) method using MEGA X software.

### 2.4 Determination of the solubilization index on PVK agar

The isolates PtWFY-1 and PtWFY-2 were preliminarily screened for their ability to solubilize insoluble phosphate sources [Ca_3_(PO_4_)_2_ and calcium phytate] on Pikovskaya's (PVK) agar. One liter (1.0 L) of PVK agar comprised the following (g/L): 10.0 g of glucose, 0.30 g of NaCl, 0.30 g of KCl, 0.5 g of (NH_4_)_2_SO_4_, 0.30 g of MgSO_4_·7H_2_O, 0.03 g of FeSO_4_·7H_2_O, 0.03 g of MnSO_4_·H_2_O, 0.03 g of Ca3(PO_4_)_2_/Al_3_(PO_4_)_2_/FePO_4_/Mg_3_(PO_4_)_2_/phosphate rock powder/calcium phytate, 5.0 g of agar, and 18.0 g of agar in 1,000 mL of distilled water (pH 7.0–7.2) (Pikovskaya, 1948). The medium was autoclaved at 121°C for 20 min. Fungal mycelium plugs (5 mm^3^), cut from the edges of actively growing colonies, were placed on PVK agar for 5 days at 28°C. Sterile PDA plugs served as controls. Three replicates were tested for each of the PtWFY-1 and PtWFY-2 isolates. The formation of visible halo zones around the microbial colonies on the plates indicated the phosphate solubilization capability of the strains. The diameter of the halo zones around the colonies and the diameter of the colonies were measured after 5 days of incubation. The phosphate solubilization index was calculated according to Premono et al. (1996).


$$\begin{array}{r} \text{Solubilization index }(\text{SI})=(\text{colony diameter}\\ +\text{halo zone diameter})\text{/colony diameter.} \end{array}$$

### 2.5 Phosphate solubilization efficiency in PVK broth

Phosphate solubilization activity testing was conducted in 50 mL centrifuge tubes containing 40 mL of PVK broth *in vitro*, which had the same composition as the PVK agar but without agar. The insoluble phosphate sources included Ca_3_(PO_4_)_2_, Al_3_(PO_4_)_2_, FePO_4_, Mg_3_(PO_4_)_2_, phosphate rock powder, and calcium phytate. The initial pH of the medium was adjusted to 7.0 before sterilization. Spore suspensions of each *Pyrenochaetopsis tabarestanensis* isolate were prepared according to Elias et al. (2016). Two percentage spore suspensions (10^7^ spores/mL) were inoculated into sterilized PVK broth. The controls consisted of 2% sterile distilled water in sterilized PVK broth. Three replicates were maintained for each test. The cultures were incubated on a rotary shaker at 28°C and 180 rpm for 7 days. The culture supernatant was aseptically collected daily from days 1 to 7. The amount of available soluble phosphorus released from the insoluble sources by the fungal strains was estimated via the molybdenum blue method at 700 nm (Ryan et al., 2001). The pH of the culture supernatant in the PVK broth was measured daily using a digital pH meter with a glass electrode (Jingci, Shanghai, Co., Ltd.).

### 2.6 Antifungal assays

The *in vitro* antibacterial activity of the PtWFY-1 and PtWFY-2 strains was also evaluated through plate confrontation assays against the plant pathogen *Magnaporthe oryzae* Guy 11, as described by Singh et al. (2013, 2014). The mediums of PtWFY-1 and PtWFY-2 served as negative controls. Three replicates were tested for each isolate. Strains demonstrating more than 50% inhibition of mycelial growth were considered promising antagonists. A total of 1.0 L of Prune agar (PA) medium consisted of 5.0 g lactose, 1.0 g yeast extract powder, 40 mL prune juice, 20 g agar, and 1,000 mL distilled water (Pikovskaya, 1948). The medium was autoclaved at 121°C for 20 min. Growth inhibition rate was calculated from mean values as: Inhibition rate (%) = (*Magnaporthe oryzae* colony diameter in Control – *Magnaporthe oryzae* colony diameter in treatment)/*Magnaporthe oryzae* colony diameter in Control × 100% (Elsharkawy et al., 2014).

### 2.7 Organic acid and phytohormone quantification

For organic acid measurement, PtWFY-1 and PtWFY-2 mycelia and spores were scraped into PVK liquid medium containing calcium phosphate to create a fungal spore suspension of 10^6^ spores/mL. This suspension was cultured on a shaking bed at 28°C and 180 rpm for 5 days and then centrifuged for 10 min at 12,000 r min^−1^ at 4°C. The supernatant was collected and stored at −80°C for further measurement of organic acids. The external standard method was used for the determination of organic acids. A total of 74 different organic acids were screened. Nine kinds of organic acids, oxalic acid, citric acid, tartaric acid, formic acid, malonic acid, acetic acid, maleic acid, aconitate, and propionic acid, were measured on a Thermo U3000 HPLC platform. The remaining organic acids were detected via MetWare on the AB Sciex QTRAP 6500 LC-MS/MS platform. Standards for the nine acids were prepared in ddH_2_O and diluted to concentrations of 0.1, 0.2, 0.5, 0.8, 1, 5, 10, 15, 20, 50, 80, 100, 150, and 200 μg/mL. The remaining standards were prepared in methanol to 1 mg/mL and diluted to concentrations of 0.01, 0.02, 0.05, 0.1, 0.2, 0.5, 1, 2, 5, 10, 20, 50, 100, 200, 500, 1,000, 5,000, and 10,000 ng/mL. Standard curves were drawn with the concentration of the external standard as the abscissa and the peak area as the ordinate. The concentration of each acid in the samples was calculated by substituting the integral peak areas into the linear equation of the standard curve. The types and standard curves of the standards are listed in Supplementary Table S3.

For phytohormone measurement, PtWFY-1 and PtWFY-2 spores were scraped into PDB liquid medium to create a fungal spore suspension of 10^6^ spores/mL. The formula of the PDB liquid medium was the same as that of the PDA medium without agar. The suspension was incubated on a shaker at 28°C and 180 rpm for 15 days and then centrifuged for 10 min at 4°C, 12,000 r min^−1^. The supernatant was stored at −80°C until further use. The external standard method and internal standard correction were applied for the quantification of target phytohormones. A total of 88 kinds of phytohormones were screened. Standards were prepared in methanol at a concentration of 1 mg/mL and then diluted to concentrations of 0.01, 0.05, 0.1, 0.5, 1, 5, 10, 50, 100, 200, and 500 ng/mL. For the measurement of L-tryptophan and salicylic acid 2-O-β-glucoside, the standard curve concentration range was adjusted to 0.2–10,000 ng/mL. Standard curves were drawn with the concentration ratio of the external standard to the internal standard as the abscissa and the peak area ratio as the ordinate. The types and standard curves of the standards are listed in Supplementary Table S4. Phytohormones were detected by MetWare (http://www.metware.cn/) based on the AB Sciex QTRAP 6500 LC-MS/MS platform. The concentration of each phytohormone in the samples was calculated by substituting the integral peak areas into the linear equation of the standard curve.

### 2.8 Greenhouse pot experiment

The effects of inoculating soils with *Pyrenochaetopsis tabarestanensis* strains on P availability, P uptake, and rice growth were assessed through pot experiments, with four replicates per treatment. Paddy soil samples containing 9 mg kg^−1^ available P and with a pH of 6.0 were air-dried, ground, sieved (< 2.0 mm), and sterilized before the experiments. Three rice seeds (Yangdao6 variety) were sown individually in pots containing 300 g of soil, and 1.0 mL of *Pyrenochaetopsis tabarestanensis* inoculant (10^6^ spores/mL) was applied. Uninoculated seeds served as controls. The plants were cultivated under greenhouse conditions with an average temperature of 28°C, a relative humidity of 50–60%, and a 12 h light and 12 h dark photoperiod with natural lighting. The soil moisture was maintained at 60% of its maximum water-holding capacity (WHC) to prevent nutrient leaching and root damage. After 30 days, three rice seedlings were harvested to measure their dry biomass and P concentration. The soil samples were analyzed for total P by the molybdenum blue method and for available P by the Olsen-P extraction method (Pansu and Gautheyrou, 2006).

### 2.9 Statistical analysis

The data were presented as the means with standard errors from three replicates. Differences between treatments were evaluated via one-way analysis of variance (ANOVA) followed by Duncan's multiple range tests, which were performed using the Statistical Package for the Social Sciences (SPSS) for Windows version 22 (SPSS, Inc., Chicago, IL, United States). Statistical significance was set at *P* ≤ 0.05. Additionally, the production of organic acids and phytohormones by the fungal strains was analyzed by cluster and heatmap analyses via the ggplots 3.0.3 package in R software version 3.2.3. UV (unit variance scaling) scaling was applied to the concentrations of organic acids and phytohormones before plotting.

## 3 Results

### 3.1 Isolation, screening, and characterization of *Pyrenochaetopsis tabarestanensis* isolates

**Figure 1:**
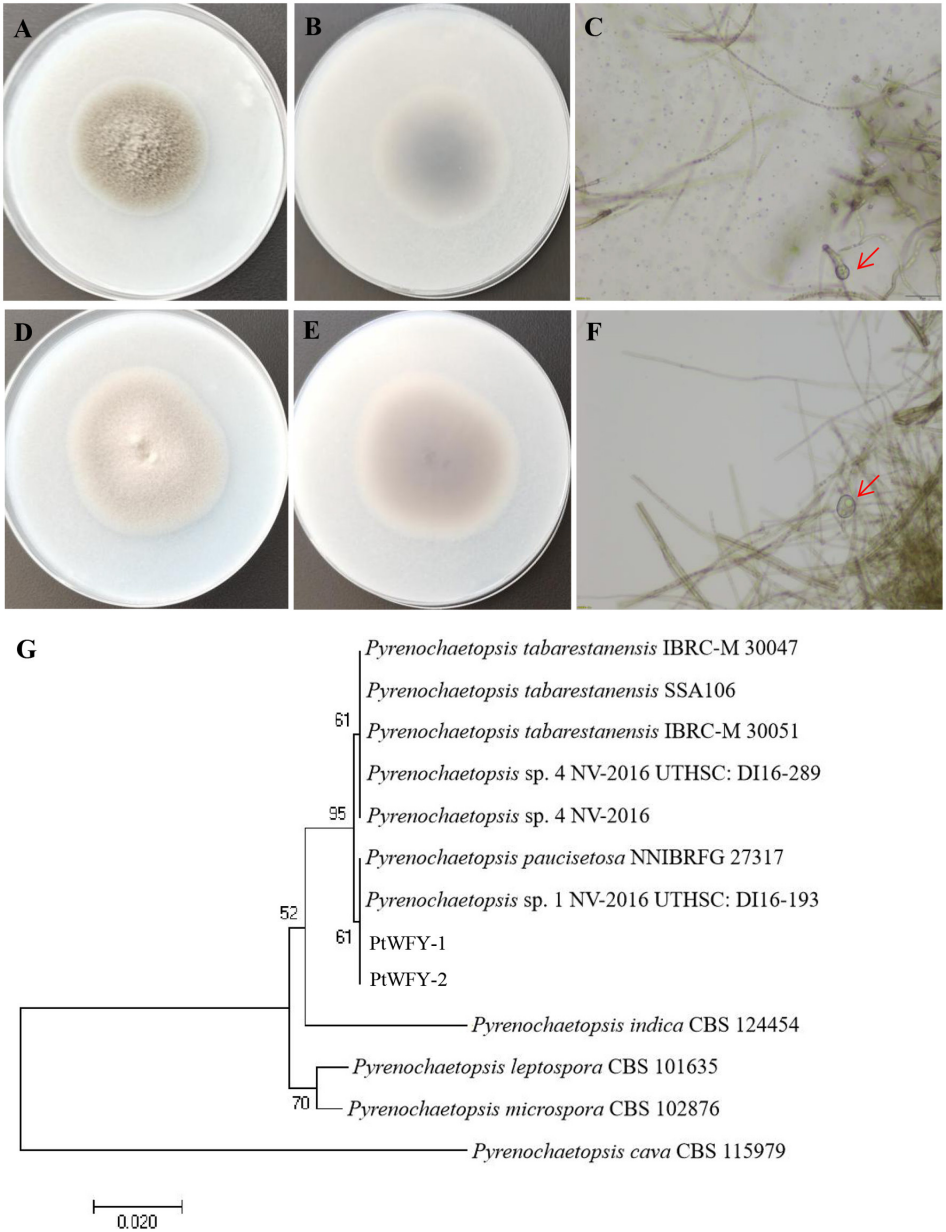
The single colony diagram of *Pyrenochaetopsis tabarestanensis* WFY-1 (PtWFY-1) and WFY-2 (PtWFY-2) on oat medium. **(A)** The frontal morphological features of PtWFY-1 strain; **(B)** The dorsal morphological features of PtWFY-1 strain; **(C)** The thick-walled spores produced by PtWFY-1; **(D)** The frontal morphological features of PtWFY-2 strain; **(E)** The dorsal morphological features of PtWFY-2 strain; **(F)** The thick-walled spores produced by PtWFY-2; **(G)** The phylogenetic tree of PtWFY-1 and PtWFY-2 strains constructed based on the homology of the ITS rDNA sequences.

Two novel strains of *Pyrenochaetopsis tabarestanensis* were isolated from the rice rhizosphere. Both fungi presented similar morphologies and growth rates. After 14 days of cultivation, both colonies were velvety and slightly flocculent, with a grayish-white center. However, the color of PtWFY-1 was grayish-brown, whereas that of PtWFY-2 was gray-brown (Figures 1A, B, D, E). The diameter of both colonies reached 50–60 mm within 14 days of culture. Thick-walled spores, characteristic of *Pyrenochaetopsis tabarestanensis*, were observed under a microscope in both colonies (Figures 1C, F). The length of the amplified ITS rDNA sequence was 442 bp for PtWFY-1 and 450 bp for PtWFY-2, with high homology (99.77%) to *Pyrenochaetopsis tabarestanensis*. The sequences of the novel isolates were deposited in the NCBI GenBank database under the accession numbers PP658459 and PP658460. On the basis of morphological characteristics and genetic analysis, both PtWFY-1 and PtWFY-2 were identified as *Pyrenochaetopsis tabarestanensis*. Additionally, the sequences of PtWFY-1 and PtWFY-2 with another eleven nucleotide sequences retrieved from the NCBI were processed for the phylogenetic tree, and their sequences revealed 61% similarity to the ITS gene sequences of *Pyrenochaetopsis tabarestanensis* 1 NV-2016 UTHSC: DI16-193 (Figure 1G).

### 3.2 Qualitative and quantitative phosphate solubilization

PtWFY-1 and PtWFY-2 demonstrated their potential for phosphate solubilization by forming clear zones on PVK agar plates containing Ca_3_(PO_4_)_2and_ calcium phytate (Supplementary Figure S1). The solubilization index (SI) of PtWFY-1 was 3.49 and 3.67 for Ca_3_(PO_4_)_2and_ calcium phytate, respectively, after 5 days of incubation (Supplementary Figures S1A, C), whereas PtWFY-2 presented SI values of 3.00 and 3.40 for Ca_3_(PO_4_)_2and_ calcium phytate, respectively (Supplementary Figures S1B, D).

**Figure 2:**
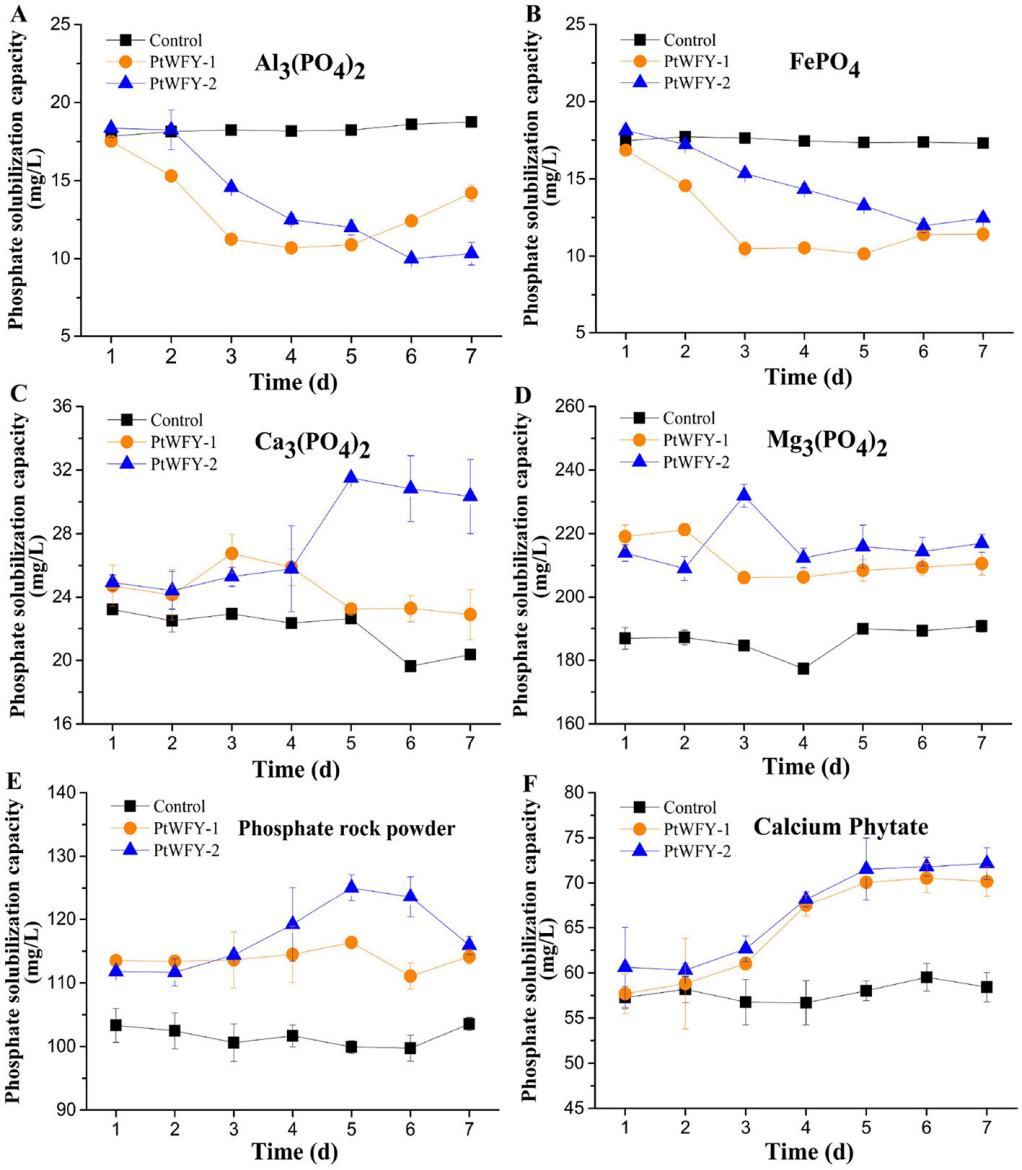
Phosphate solubilizing characteristics of *Pyrenochaetopsis tabarestanensis* WFY-1 (PtWFY-1) and WFY-2 (PtWFY-2) under PVK liquid culture conditions. **(A)** The phosphate solubilizing effect of PtWFY-1 and PtWFY-2 strains on aluminum phosphate [Al_3_(PO_4_)_2_] under PVK liquid culture conditions; **(B)** The phosphate solubilizing effect of PtWFY-1 and PtWFY-2 strains on iron phosphate (FePO_4_) under PVK liquid culture conditions; **(C)** The phosphate solubilizing effect of PtWFY-1 and PtWFY-2 strains on calcium phosphate [Ca_3_(PO_4_)_2_] under PVK liquid culture conditions; **(D)** The phosphate solubilizing effect of PtWFY-1 and PtWFY-2 strains on magnesium phosphate [Mg_3_(PO_4_)_2_] under PVK liquid culture conditions; **(E)** The phosphate solubilizing effect of PtWFY-1 and PtWFY-2 strains on phosphate rock powder under PVK liquid culture conditions; **(F)** The phosphate solubilizing effect of PtWFY-1 and PtWFY-2 strains on calcium phytate under PVK liquid culture conditions. Phosphorus concentrations were quantified from PVK broth at 1, 2, 3, 4, 5, 6, and 7 days. Control: PVK broth without PtWFY-1 or PtWFY-2 inoculation; PtWFY-1: PVK broth with PtWFY-1 inoculation; PtWFY-2: PVK broth with PtWFY-2 inoculation. Means and standard errors from three replicates are shown.

PtWFY-1 and PtWFY-2 were able to dissolve Ca_3_(PO_4_)_2_, Mg_3_(PO_4_)_2_, phosphate rock powder, and calcium phytate in PVK broth but not Al_3_(PO_4_)_2_ or FePO_4_ (Figure 2). When Ca_3_(PO_4_)_2_ was utilized as the P source, the soluble P concentration increased by 21.8% (day 3) for PtWFY-1 and by 43.2% (day 5) for PtWFY-2 relative to the uninoculated control (Figure 2A). Similarly, for Mg_3_(PO_4_)_2_, the soluble P concentration increased on day 2 for PtWFY-1 (19.6%) and day 3 for PtWFY-2 (25.4%) relative to the uninoculated control (Figure 2B). With phosphate rock powder as the P source, the soluble P concentrations in the culture medium of PtWFY-1 and PtWFY-2 increased by 16.4% and 25.0%, respectively, on day 5 compared with those in the uninoculated control (Figure 2C). In the case of calcium phytate, the soluble P concentration in the culture medium of PtWFY-1 and PtWFY-2 increased 21.6% and 24.5%, respectively, on days 6 and 7 relative to that of the uninoculated control (Figure 2D).

### 3.3 Contribution of the PtWFY-1 and PtWFY-2 strains to resistance against *Magnaporthe oryzae*, plant phosphorus absorption, and phosphorus availability in soil

**Figure 3:**
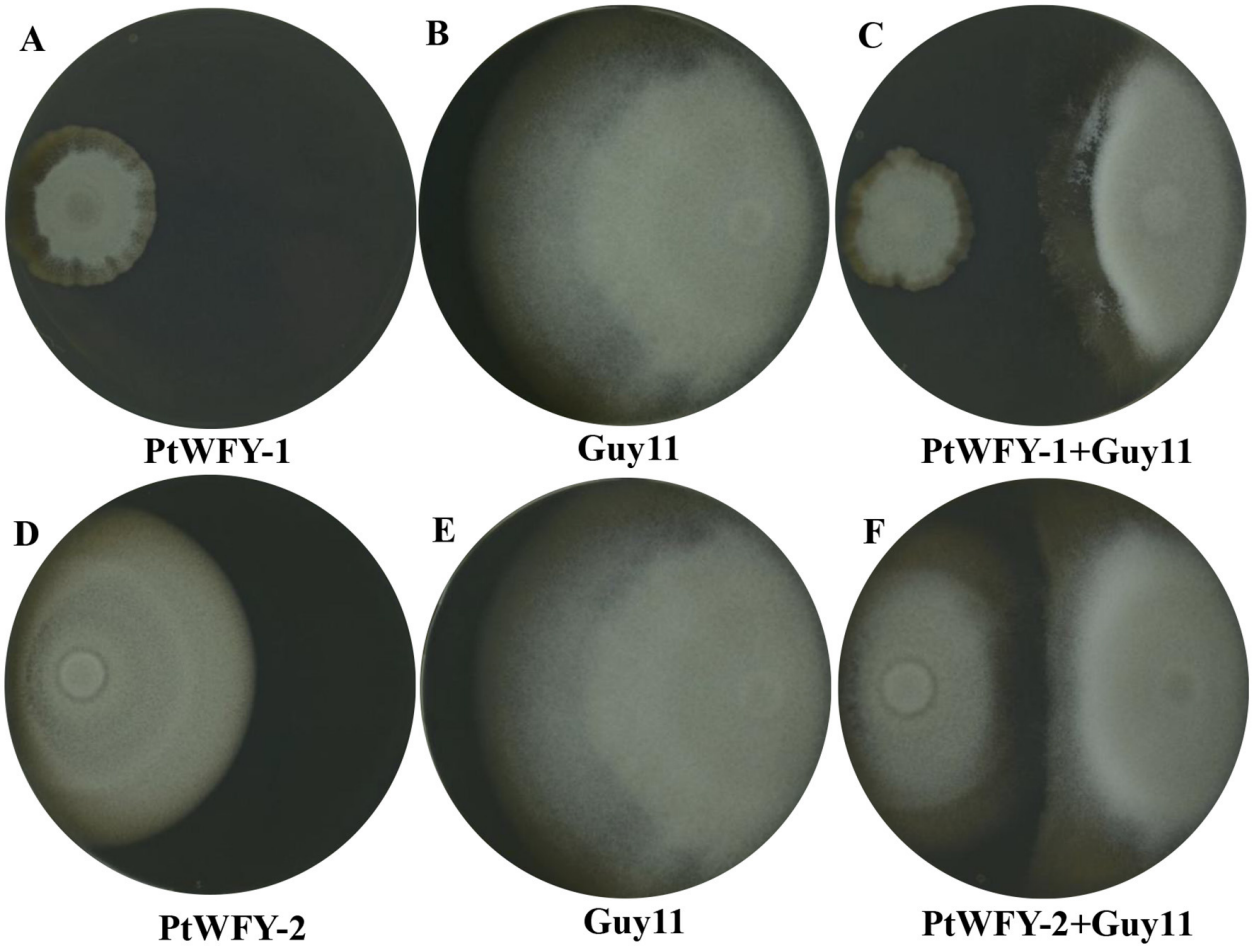
The inhibitory effect of *Pyrenochaetopsis tabarestanensis* WFY-1 (PtWFY-1) and WFY-2 (PtWFY-2) on *Magnaporthe oryzae* (Guy11). **(A)** The morphology of PtWFY-1 colony; **(B)** The morphology of normal-growing colony of Guy11; **(C)** The morphology of PtWFY-1 + Guy11; **(D)** The morphology of PtWFY-2 colony; **(E)** The morphology of normal-growing colony of Guy11; **(F)** The morphology of PtWFY-2 + Guy11. Guy11 referred to a physiological race of *Magnaporthe oryzae*.

The PtWFY-1 and PtWFY-2 strains demonstrated antibacterial activity against the plant pathogen *Magnaporthe oryzae* Guy 11 on PA agar plates. Initially, the diameters of the PtWFY-1 and PtWFY-2 strains were 3.2 cm and 7.6 cm, respectively, and the diameter of *Magnaporthe oryzae* Guy 11 was 6.38 cm (Figures 3A, B, D, E). Upon placement of the PtWFY-1 and PtWFY-2 strains at opposite positions, the diameter decreased to 2.57 and 2.88 cm, respectively (Figures 3C, F). The inhibition rates of the PtWFY-1 and PtWFY-2 strains were 59.7% and 54.9%, respectively. Compared with the PtWFY-2 strain, the PtWFY-1 strain exhibited a relatively greater inhibitory effect on *Magnaporthe oryzae*. This characteristic makes the PtWFY-1 and PtWFY-2 strains desirable candidates for antibacterial purposes.

**Figure 4:**
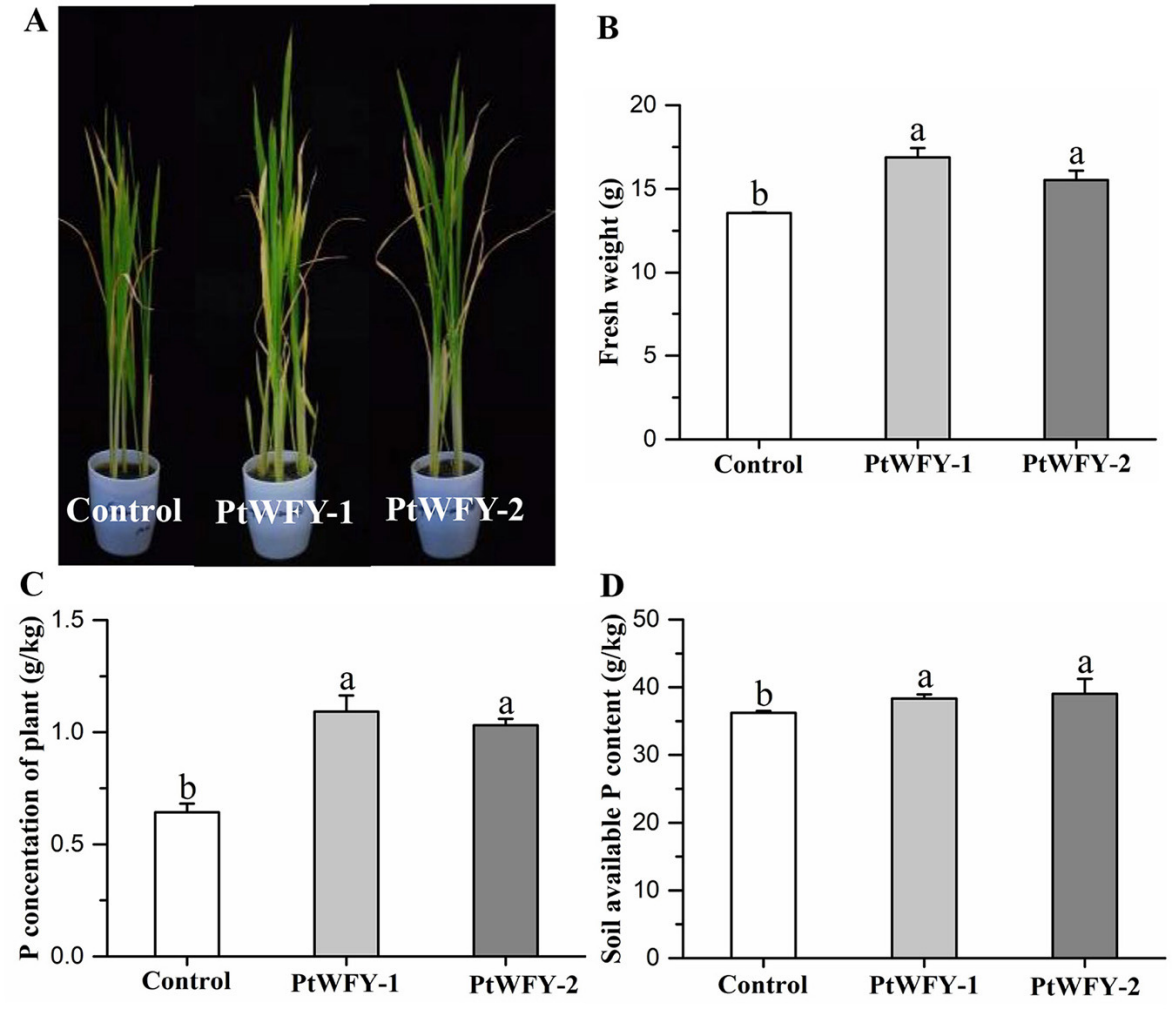
The promotion of rice phosphorus uptake and soil phosphorus solubilization by *Pyrenochaetopsis tabarestanensis* WFY-1 (PtWFY-1) and WFY-2 (PtWFY-2). **(A)** Rice growth status for inoculation of PtWFY-1 and PtWFY-2 strains vs. control; **(B)** The effect of control vs. PtWFY-1 and PtWFY-2 strains on the fresh weight of the rice plant; **(C)** The effect of control vs. PtWFY-1 and PtWFY-2 on phosphorus concentration of the rice plant; **(D)** The effect of control vs. PtWFY-1 and PtWFY-2 on the soil available phosphorus content. Control represent the no inoculation, while PtWFY-1 and PtWFY-2 represent inoculation treatments. Means and standard errors from four replicates are shown. Different lower case letters indicate significant differences at the *p* < 0.05 level.

Inoculation with the PtWFY-1 and PtWFY-2 strains in the soil treatments resulted in a significant increase in the growth of the rice seedlings, plant P content, and total soil P (Figure 4). Compared with the uninoculated control, PtWFY-1 and PtWFY-2 inoculation resulted in 24.5% and 14.5% increases in fresh weight, respectively (Figure 4B). The total P concentration of the rice plants significantly increased by 69.8% and 60.3% in response to PtWFY-1 and PtWFY-2 inoculation, respectively (Figure 4C). Additionally, the available P content in the soil significantly increased by 5.80% and 7.70% following PtWFY-1 and PtWFY-2 inoculation, respectively (Figure 4D).

### 3.4 Organic acids secreted by the PtWFY-1 and PtWFY-2 strains

There was a significant decline in the pH of the PtWFY-1 and PtWFY-2 culture medium, indicating the secretion of organic acids by these strains to dissolve insoluble phosphate (Supplementary Figure S2). Compared with those in the control treatment, the pH levels of Ca_3_(PO_4_)_2_, Al_3_(PO_4_)_2_, FePO_4_, Mg_3_(PO_4_)_2_, and phosphate rock powder broth inoculated with both strains decreased by 5.0%, 36.5%, 25.5%, 7.4%, and 17.4%, respectively, around day 7 (Supplementary Figures S2A–E). Additionally, the pH of the calcium phytate broth decreased by 33.9% and 23.2% after PtWFY-1 and PtWFY-2 inoculation, respectively, around day 7 (Supplementary Figure S2F).

**Figure 5:**
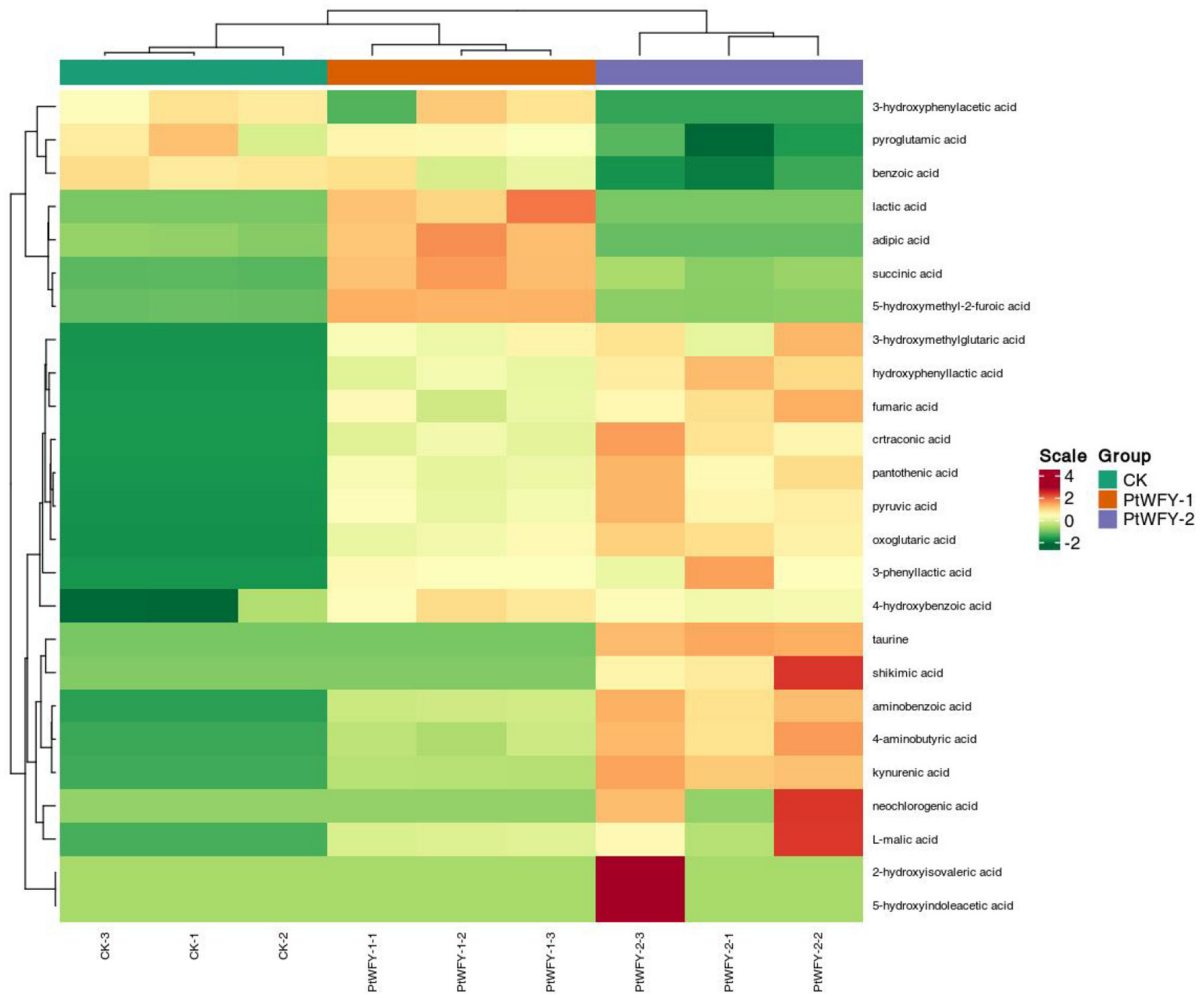
Cluster and heat-map analysis of the concentration of organic acids during *in vitro* solubilization of inorganic P sources by PtWFY-1 and PtWFY-2 for tricalcium phosphate. The horizontal indicates the sample name, and the vertical indicates the metabolite information. Group indicates the treatment. Green and red indicate normalized concentration gradient of concentration of organic acids from low to high, respectively. The clustering line on the left side of the figure is the metabolite clustering line, and the clustering line on the top of the figure is the sample clustering line. Control represent the no inoculation, while PtWFY-1 and PtWFY-2 represent inoculation treatments.

A total of 26 types of organic acids were produced by the PtWFY-1 isolate, including oxoglutaric acid (1,900.03 mg L^−1^), acetic acid (1,478.47 mg L^−1^), pyruvic acid (685.90 mg L^−1^), citric acid (579.11 mg L^−1^), malonic acid (306.97 mg L^−1^), formic acid (250.13 mg L^−1^), lactic acid (239.22 mg L^−1^), succinic acid (205.17 mg L^−1^), aconitate (175.60 mg L^−1^), tartaric acid (166.49 mg L^−1^), citraconic acid (132.73 mg L^−1^), 5-hydroxymethyl-2-furoic acid (129.47 mg L^−1^), oxalic acid (111.00 mg L^−1^), L-malic acid (92.81 mg L^−1^), and other organic acids (Supplementary Table S1). PtWFY-2 broth also contained 26 types of organic acids, with 23 types being the same as those observed in PtWFY-1 broth, including oxoglutaric acid, pyruvic acid, succinic acid, 5-hydroxymethyl-2-furoic acid, citraconic acid, L-malic acid, 4-aminobutyric acid, fumaric acid, pantothenic acid, kynurenic acid, aminobenzoic acid, 4-hydroxybenzoic acid, 3-phenyllactic acid, hydroxyphenyllactic acid, 3-hydroxymethylglutaric acid, oxalic acid, citric acid, tartaric acid, formic acid, malonic acid, acetic acid, aconitate, and propionic acid (Supplementary Table S1). The composition of the PtWFY-2 broth included oxoglutaric acid (2,441.67 mg L^−1^), acetic acid (1,519.18 mg L^−1^), pyruvic acid (888.30 mg L^−1^), citric acid (867.65 mg L^−1^), formic acid (328.86 mg L^−1^), malonic acid (289.56 mg L^−1^), citraconic acid (199.87 mg L^−1^), aconitate (194.69 mg L^−1^), L-malic acid (154.17 mg L^−1^), tartaric acid (144.38 mg L^−1^), oxalic acid (121.25 mg L^−1^), 4-aminobutyric acid (79.10 mg L^−1^), succinic acid (59.90 mg L^−1^), and other organic acids (Supplementary Table S1). The PtWFY-1 strain uniquely secreted adipic acid, maleic acid, and lactic acid, whereas the PtWFY-2 strain specifically produced shikimic acid, taurine, and neochlorogenic acid (Figure 5). Furthermore, oxoglutaric acid, acetic acid, pyruvic acid, and citric acid were identified as the primary organic acids produced by both the PtWFY-1 and PtWFY-2 isolates compared with the control (Supplementary Table S1).

### 3.5 Phytohormones secreted by the PtWFY-1 and PtWFY-2 strains

**Figure 6:**
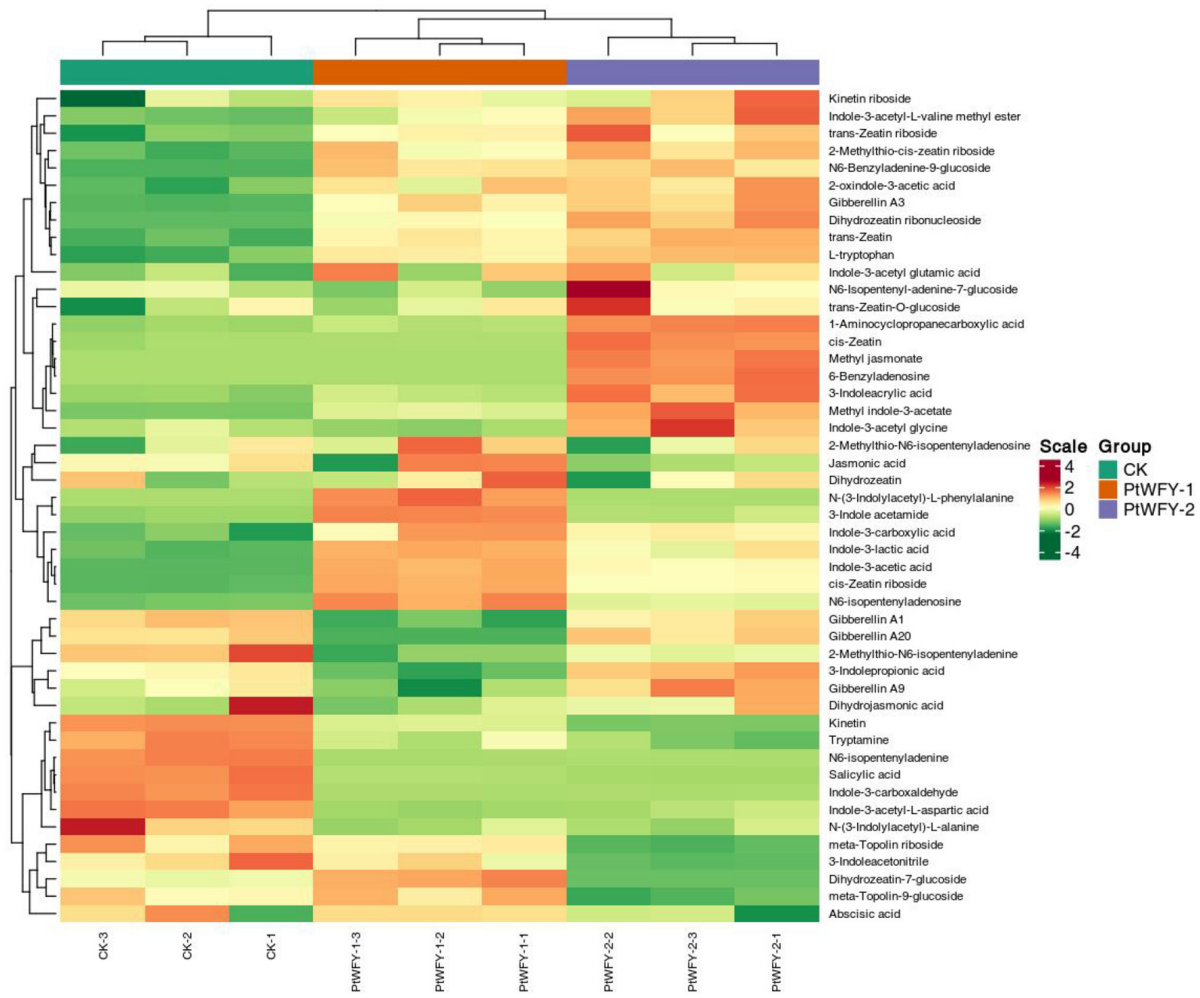
Cluster and heat-map analysis of the concentration of phytohormones produced by PtWFY-1 and PtWFY-2. The horizontal indicates the sample name, and the vertical indicates the phytohormones information. Group indicates the treatment. Green and red indicate normalized concentration gradient of concentration of phytohormones from low to high, respectively. The clustering line on the left side of the figure is the phytohormones clustering line, and the clustering line on the top of the figure is the sample clustering line. Control represent the no inoculation, while PtWFY-1 and PtWFY-2 represent inoculation treatments.

In the PDB broth of the PtWFY-1 isolate, 28 types of phytohormones were identified, whereas 30 types were observed in the PDB broth of the PtWFY-2 isolate (Supplementary Table S2). Among these, 23 types of phytohormones, including L-tryptophan, 1-aminocyclopropanecarboxylic acid, Indole-3-acetic acid, 2-oxindole-3-acetic acid, Indole-3-lactic acid, N6-isopentenyladenosine, Indole-3-carboxylic acid, 3-indole acetamide, cis-zeatin riboside, 2-methylthio-N6-isopentenyladenosine, Gibberellin A3, dihydrozeatin ribonucleoside, trans-zeatin-O-glucoside, 3-indoleacrylic acid, trans-zeatin, Indole-3-acetyl glutamic acid, kinetin riboside, 2-methylthio-cis-zeatin riboside, trans-zeatin riboside, methyl indole-3-acetate, N6-benzyladenine-9-glucoside, cis-zeatin, and Indole-3-acetyl-L-valine methyl ester (Supplementary Table S2), were common to both strains. The predominant composition of the PtWFY-1 broth was 84.68% L-tryptophan (3,541.57 mg/L), 13.38% 1-aminocyclopropanecarboxylic acid (559.54 mg/L), and 1.35% indole-3-acetic acid (56.60 mg/L). Similarly, the primary composition of the PtWFY-2 broth was 83.46% L-tryptophan (3,769.21 mg/L), 15.24% 1-aminocyclopropanecarboxylic acid (688.35 mg/L), and 0.82% indole-3-acetic acid (37.00 mg/L) (Supplementary Table S2). The unique phytohormone types produced by the PtWFY-1 strain included N-(3-indolylacetyl)-L-phenylalanine, meta-topolin-9-glucoside, dihydrozeatin, and jasmonic acid, whereas gibberellin A20, methyl jasmonate, 6-benzyladenosine, indole-3-acetyl glycine, N6-isopentenyl-adenine-7-glucoside, 3-indolepropionic acid, and gibberellin A9 were specific to the PtWFY-2 strain (Figure 6). L-tryptophan was the principal phytohormone produced by both the PtWFY-1 and PtWFY-2 isolates (Supplementary Table S2).

## 4 Discussion

### 4.1 Two *P. tabarestanensis* strains isolated from the rice rhizosphere

*Pyrenochaetopsis* species are ubiquitous in nature and have been isolated from various ecosystems, such as freshwater, rice paddies, and ornamental boxwood (Papizadeh et al., 2017; Wang et al., 2019; Magana-Duenas et al., 2021; Špetík et al., 2021). However, *Pyrenochaetopsis tabarestanensis* strains have not previously been reported to be isolated from the rice rhizosphere (Papizadeh et al., 2017; Chen et al., 2020). In this study, we successfully isolated two *P. tabarestanensis* strains, and for the first time, these strains were obtained from the rice rhizosphere. *Pyrenochaetopsis* species often exhibit overlapping features, making it challenging to differentiate between genera (Papizadeh et al., 2017). Therefore, the identities of the PtWFY-1 and PtWFY-2 strains in this study were determined by a polyphasic approach, including morphological observations, sequencing of ITS regions, and phylogenetic analysis. The presence of thick-walled spores serves as the basis for identifying these two strains as *P. tabarestanensis* (Valenzuela-Lopez et al., 2018). Additionally, ITS rRNA sequence analysis revealed 99.77% similarity with known *P. tabarestanensis* strains. Phylogenetic analysis further supported their taxonomic classification. The isolation and identification of these *P. tabarestanensis* strains from the rice rhizosphere provide a foundation for further exploration of the functions of *Pyrenochaetopsis* species.

### 4.2 Phosphate solubilization ability of the PtWFY-1 and PtWFY-2 strains

The PtWFY-1 and PtWFY-2 strains exhibited solubilization of various phosphate sources, including Ca_3_(PO_4_)_2_, Mg_3_(PO_4_)_2_, phosphate rock powder, and calcium phytate, while showing no solubilization of Al_3_(PO_4_)_2and_ FePO_4_ (Figure 2), which was consistent with previous findings (Satyaprakash et al., 2017). Previous studies have indicated that Ca_3_(PO_4_)_2_ starts to dissolve when the pH drops below 4.0, whereas FePO_4_ remains insoluble until the pH decreases to 2.5 or lower (Jiang et al., 2020). In this study, we observed that Ca_3_(PO_4_)_2_ began to dissolve when the pH was below 6.0, thereby expanding the pH range for the dissolution of insoluble Ca_3_(PO_4_)_2_. Furthermore, the pH of the PVK broth containing Al_3_(PO_4_)_2and_ FePO_4_ remained above 3.0 on day 7 (Supplementary Figures S2A, B), resulting in the insolubilization of Al_3_(PO_4_)_2and_ FePO_4_. Since calcium predominates were the primary phosphate fertilizer in paddy soils, the solubility of Ca_3_(PO_4_)_2_ indicated that the PtWFY-1 and PtWFY-2 strains were suitable phosphate-solubilizing fungal fertilizer candidates for application in paddy fields.

In this investigation, the PtWFY-1 and PtWFY-2 strains were found to possess greater phosphate solubilizing abilities toward Ca_3_(PO_4_)_2_ than Mg_3_(PO_4_)_2_, phosphate rock powder, and calcium phytate. As a result, these strains are promising candidates for increasing P solubilization, particularly in paddy soils where calcium predominates as the primary phosphate fertilizer. However, compared with the phosphate solubilization efficiency of *Aspergillus* and *Penicillium* species isolated from soil samples in previous studies, the PtWFY-1 and PtWFY-2 strains exhibited lower efficacy (Saxena et al., 2013; Xie et al., 2019). This finding cannot deny their value in paddy fields because of their other ecological functions, such as rice growth-promoting effects and resistance against *Magnaporthe oryzae*. In the meantime, this suggests that factors such as application conditions (e.g., concentration, interactions with other microorganisms) and environmental variables (including soil element content, biotic, and abiotic stresses) should be carefully considered when employing these fungal strains in practical applications.

### 4.3 Mechanisms involved in phosphate solubilization by the PtWFY-1 and PtWFY-2 strains

In this study, LC-MS/MS and HPLC analysis results (Supplementary Tables S1, S2) demonstrated that the PtWFY-1 and PtWFY-2 strains secreted primarily oxoglutaric acid, acetic acid, citric acid, and pyruvic acid, with most of these acids being involved in the Krebs cycle (Li et al., 2016). These findings partially align with previous research indicating that oxalic acid and citric acid are the main organic acids produced by PSF (Islam et al., 2019). Citric acid is an intermediate product of the Krebs cycle, and its secretion has been described in many species, such as *Aspergillus, Penicillium*, and *Eupenicillium* (Adhikari and Pandey, 2019; de Oliveira Mendes et al., 2014). According to earlier studies, acetic acid generated by the PtWFY-1 and PtWFY-2 strains can enter the Krebs cycle via conversion to acetyl-CoA, similar to its secretion by *Penicillium oxalicum* (Yang et al., 2022). Oxoglutaric acid, an essential intermediate in the microbial Krebs cycle, acts as a critical link between intracellular carbon and nitrogen metabolism and is positioned after isocitric acid and before succinyl coenzyme A (Miller and Smith-Magowan, 1990). The synthesis of oxoglutaric acid by phosphorus-soluble fungi has rarely been reported, suggesting that its secretion is an innovative mechanism for dissolving phosphate substrates (Zhang et al., 2022). Pyruvic acid, a key metabolite within cells, connects the central metabolic pathways of glycolysis and the Krebs cycle, linking to various branching metabolic pathways (Roosterman and Cottrell, 2021). Its involvement in phosphate solubilization has been observed in species of *Aspergillus, Penicillium*, and *Talaromyces*, highlighting its importance as another major component of phosphorus dissolution (Brazhnikova et al., 2022; Zúñiga-Silgado, 2020). Our findings also revealed no apparent correlation between the soluble phosphorus content and total acid production (data not shown). This observation is consistent with Jiang et al. (2018) research, suggesting variability among PSM strains in terms of organic acid types and concentrations, which may contribute to diverse solubilization mechanisms.

### 4.4 The capacity and mechanisms by which the PtWFY-1 and PtWFY-2 strains increase rice P uptake

Liquid medium experiments are commonly employed to assess the ability of microorganisms to solubilize P. However, these experiments may not directly mirror the impact of these microbes on P availability in soil and subsequent plant uptake (Kanse et al., 2015; López et al., 2020). Consequently, this study sought confirmatory evidence regarding the efficacy of *P. tabarestanensis* as an inoculant in soil and its influence on P availability and nutrition in rice seedlings through greenhouse experiments (Figure 4). Similar capabilities have been documented for other fungi, such as *Westerdykella, Trichoderma, Rhizopus, Lasiodiplodia* (Srivastava et al., 2012), *Trichoderma harzianum* (Chagas et al., 2017), and *Penicillium bilaii* (Geethalakshmi et al., 2017). In this study, since the inoculated plants did not receive additional soluble P, it is plausible that the organic acids produced by the two strains played a role in desorbing P from the soil minerals. Organic acids can increase P accessibility in soil by excreting protons to reduce the soil pH, by forming complexes with cations on the surface of soil minerals, or by blocking P absorption sites on soil particles (Behera et al., 2017; Lobo et al., 2019; López et al., 2020).

P is an essential element for rice growth and is directly involved in the metabolism of sugars and proteins. An increase in soil available P is helpful for the rice growth process and development (Rose et al., 2013). The improvement in rice growth parameters, such as plant dry weight, induced by the PtWFY-1 and PtWFY-2 strains also led to a notable increase in rice phosphorus absorption. In addition to P solubilization, phytohormone production has been proposed as a significant factor in promoting rice growth (Fitriatin et al., 2022). In this study, the PtWFY-1 and PtWFY-2 strains secreted primarily L-tryptophan, which emerged as the major phytohormone (Figure 6; Supplementary Table S2). Auxin, a well-known phytohormone consisting mainly of indole acetic acid (IAA), is derived predominantly from L-tryptophan through the indole-3-pyruvic acid pathway (Ahmad et al., 2005). Auxin plays a pivotal role in regulating various agronomic traits in rice, such as root architecture, tillering, inflorescence architecture, seed quality, and stress responses (Wang et al., 2018). Therefore, the secretion of L-tryptophan represents another significant trait of the PtWFY-1 and PtWFY-2 strains that contributes to the increase in rice phosphorus uptake.

The strains PtWFY-1 and PtWFY-2 displayed plant growth-promoting characteristics, including their ability to combat *Magnaporthe oryzae. Magnaporthe oryzae* is notably damaging, as it causes rice blast, a highly destructive disease affecting rice plants throughout their growth stages. It leads to annual losses ranging from 10 to 30% in various regions where rice is cultivated (Law et al., 2017). Similar antagonistic effects against *M. oryzae* have been observed in other fungi, such as *Cladosporium, Penicillium, Talaromyces, and Aspergillus* (Chaibub et al., 2020; Landum et al., 2016). The findings from this study suggest that *P. tabarestanensis* holds promise as a biocontrol agent against *M. oryzae*. However, further research is warranted to elucidate the precise mechanisms by which *P. tabarestanensis* inhibits *M. oryzae*.

P is a pressing concern in paddy fields because of its limited availability and effectiveness in agriculture (Bünemann, 2015; Dash and Dangar, 2017). The exploration of PSMs has emerged as an innovative approach to address this issue, offering significant benefits for both environmental sustainability and agricultural productivity (Mahanty et al., 2017). The discovery of the PtWFY-1 and PtWFY-2 strains, which demonstrate phosphate solubilization and enhance plant P uptake in soils, highlights the potential of *P*. *tabarestanensis* for biofertilization strategies in paddy soils lacking available phosphorus. Future research directions should include (i) experimenting with various P sources in soil; (ii) examining the effects on soil-available P and rice P uptake across different growth stages; (iii) investigating the detailed mechanisms involved in rice P uptake; and (iv) determining the optimal application conditions (e.g., concentration, interaction with other microorganisms) and considering environmental factors (e.g., soil element content, biotic and abiotic stresses).

## 5 Conclusions

The solubilization of insoluble phosphate has not been documented previously for *P. tabarestanensis*. This research isolated and identified two novel strains of *P. tabarestanensis* from the rice rhizosphere, confirming their role as PSMs. These strains effectively solubilized phosphate, increased soil phosphorus availability, and improved rice phosphorus nutrition. The secretion of oxoglutaric acid, acetic acid, citric acid, and pyruvic acid was identified as a mechanism responsible for dissolving insoluble phosphate in the rhizosphere, increasing the availability of, phosphorus for plant uptake. L-tryptophan production, which is a precursor substance for IAA synthesis, has been proposed as a major means of attaining growth promotion in rice, along with P absorption. The antibacterial activity against *Magnaporthe oryzae* contributed to healthy rice growth, indirectly promoting rice phosphorus absorption. These isolates show promise for field applications as innovative inoculants to enhance phosphorus use efficiency in paddy fields. Future studies should focus on the detailed molecular and functional characterization of these PSMs for practical field applications.

## Data Availability

The datasets presented in this study can be found in online repositories. The names of the repository/repositories and accession number(s) can be found in the article/Supplementary material.
