# Supplementary material for: Screening and identification of two novel phosphate-solubilizing Pyrenochaetopsis tabarestanensis strains and their role in enhancing phosphorus uptake in rice
Source: Front Microbiol. 2025 Jan 9;15:1494859. doi: 10.3389/fmicb.2024.1494859 (PMC11754195; doi:10.3389/fmicb.2024.1494859)
Supplement: Supplementary file 1 [file Data_Sheet_1.docx]

**A**

**B**


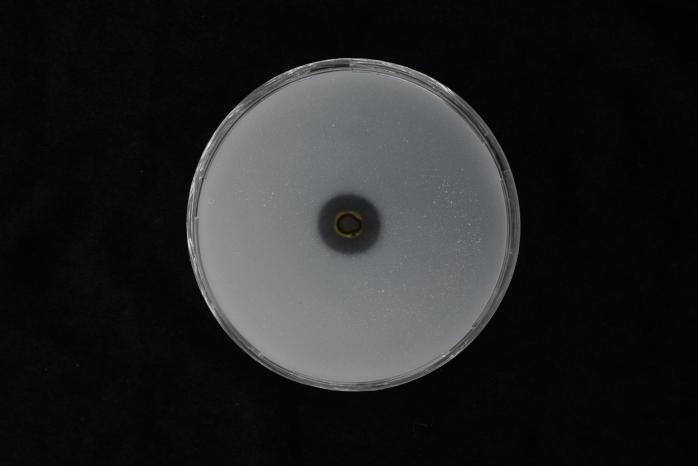

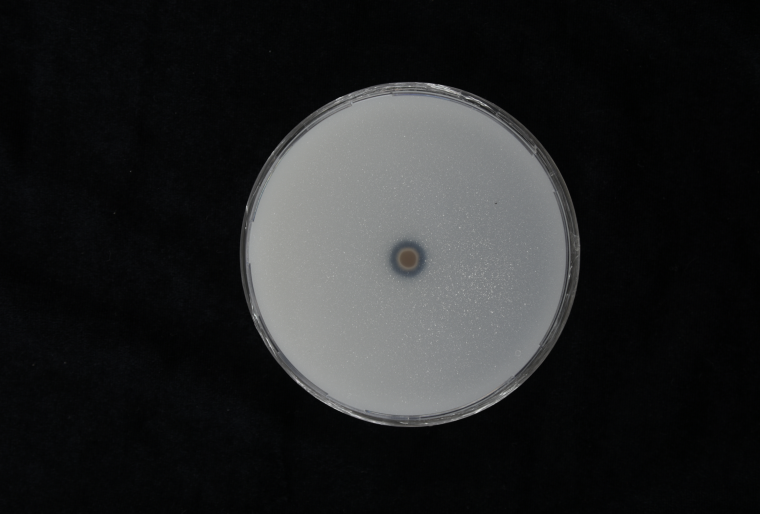


**PtWFY-1**

**PtWFY-2**


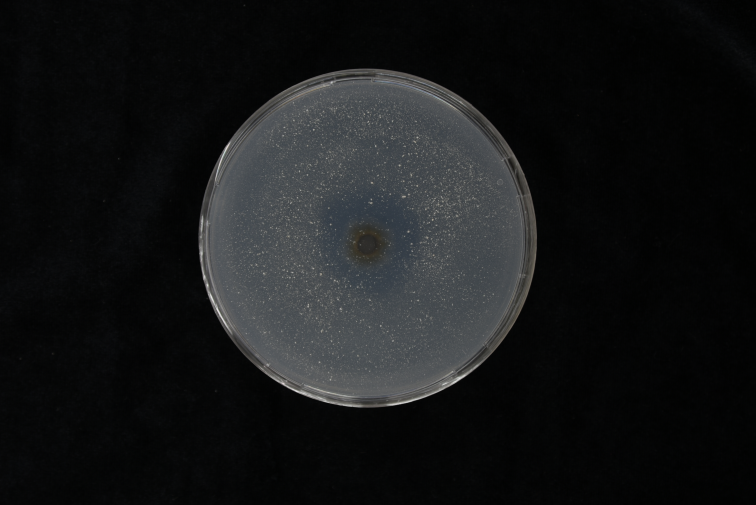

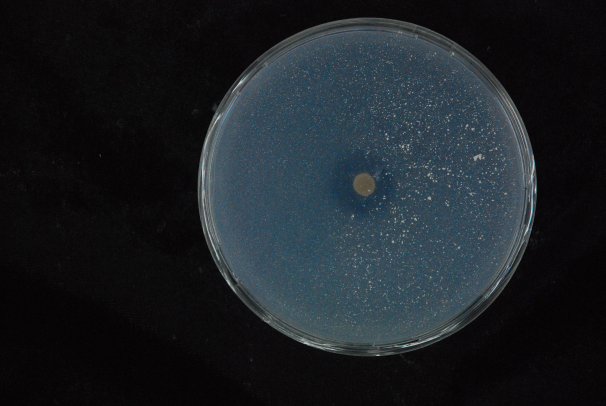


**C**

**D**

**E**

**Calcium phosphate**

**Calcium phytate**

Fig. S1 Phosphate solubilization characterization of *Pyrenochaetopsis tabarestanensis* WFY-1 (PtWFY-1) and WFY-2 (PtWFY-2) strains on PVK solid medium. A The phosphate solubilizing circle produced by PtWFY-1 strain on PVK solid medium supplemented with calcium phosphate (Ca_3_(PO_4_)_2_) (incubated for 72 h); B The phosphate solubilizing circle produced by PtWFY-2 strain on PVK solid medium supplemented with calcium phosphate (Ca_3_(PO_4_)_2_) (incubated for 72 h); C The phosphate solubilizing circle produced by PtWFY-1 strain on PVK solid medium supplemented with calcium phytate (incubated for 72 h); D The phosphate solubilizing circle produced by PtWFY-2 strain on PVK solid medium supplemented with calcium phytate (incubated for 72 h); E The phosphate solubilizing capacity of PtWFY-1 and PtWFY-2 strains on calcium phosphate and calcium phytate under PVK solid medium conditions.


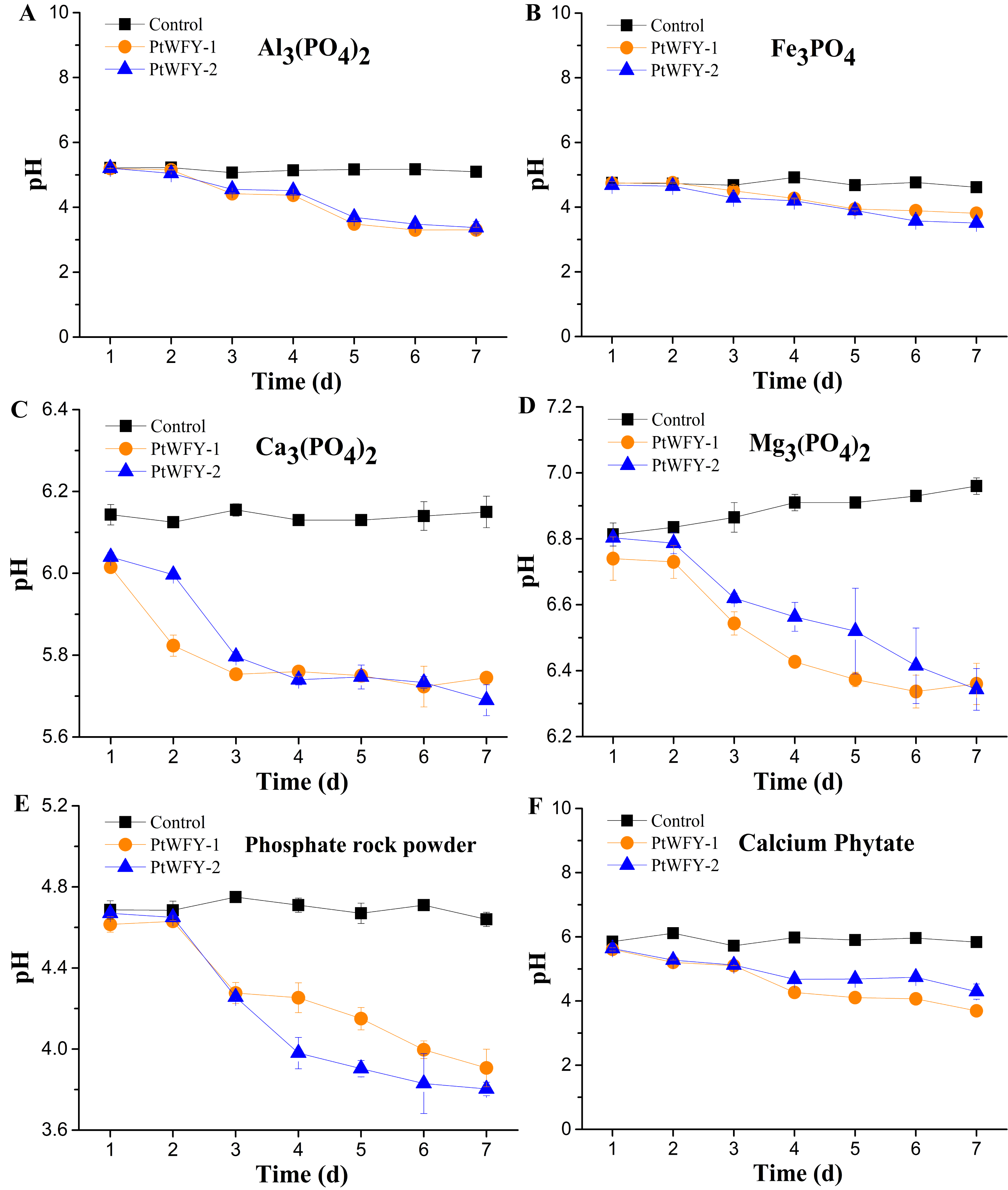


Fig. S2 The pH change of fermentation broth of *Pyrenochaetopsis tabarestanensis* WFY-1 (PtWFY-1) and WFY-2 (PtWFY-2). A The pH change of PtWFY-1 and PtWFY-2 strains in aluminium phosphate (Al_3_(PO_4_)_2_) fermentation broth; B The pH change of PtWFY-1 and PtWFY-2 strains in iron phosphate (FePO_4_) fermentation broth; C The pH change of PtWFY-1 and PtWFY-2 strains in calcium phosphate (Ca_3_(PO_4_)_2_) fermentation broth; D The pH change of PtWFY-1 and PtWFY-2 strains in magnesium phosphate (Mg_3_(PO_4_)_2_) fermentation broth; E The pH change of PtWFY-1 and PtWFY-2 strains in phosphate rock powder fermentation broth; F The pH change of PtWFY-1 and PtWFY-2 strains in calcium phytate fermentation broth. pH was quantified from PVK broth at 1, 2, 3, 4, 5, 6 and 7 days. Control: PVK broth without PtWFY-1 or PtWFY-2 inoculation; PtWFY-1: PVK broth with PtWFY-1 inoculation; PtWFY-2: PVK broth with PtWFY-2 inoculation. Different letters indicate significant differences at the *P* < 0.05 level. Means and standard errors from three replicates are shown.


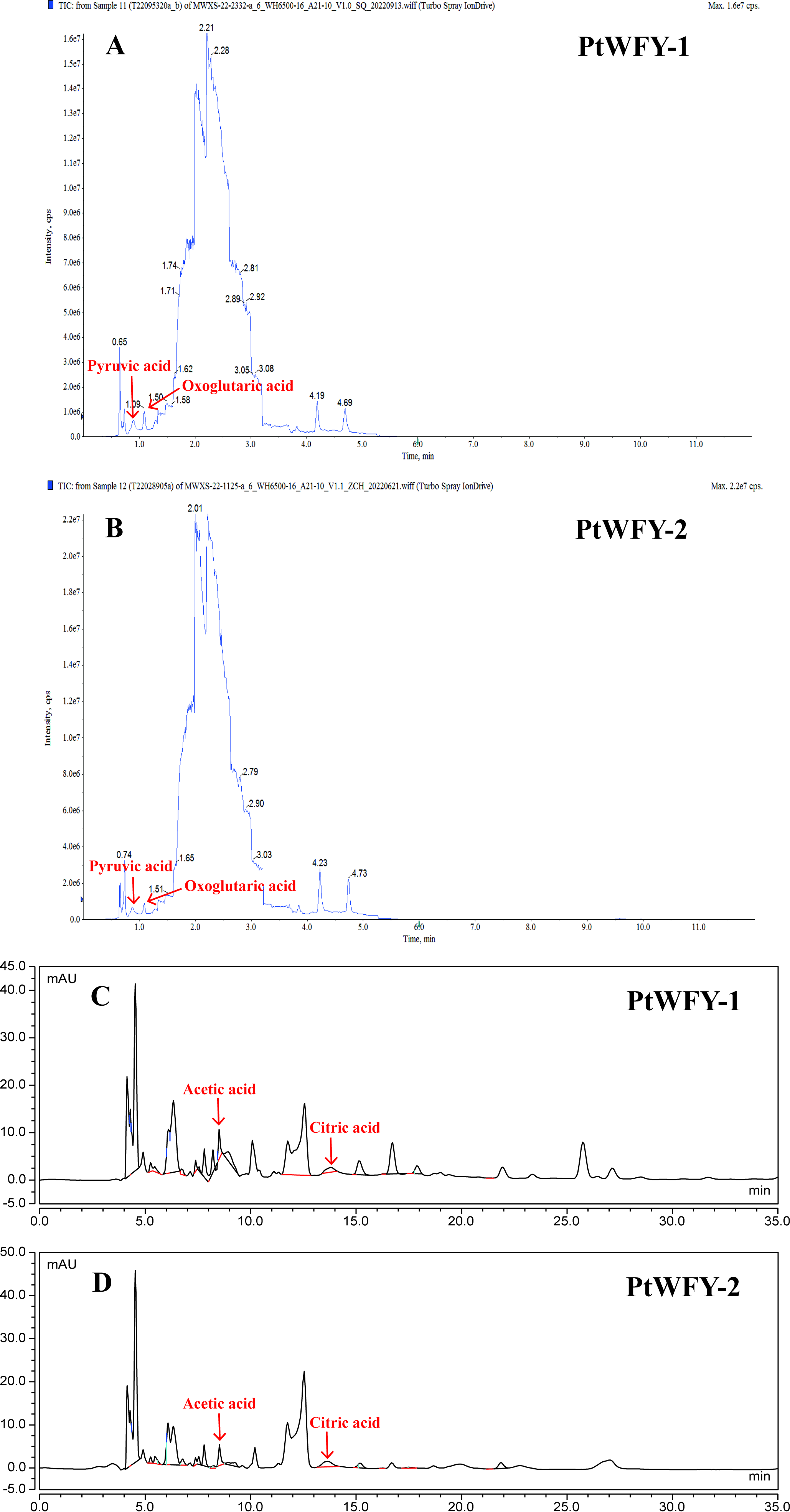


Fig. S3 The spectra of organic acids secreted by *Pyrenochaetopsis tabarestanensis* WFY-1 (PtWFY-1) and WFY-2 (PtWFY-2). A The spectra of PtWFY-1 strain in Ca_3_(PO_4_)_2_ fermentation broth detected by LC-MS/MS; B The spectra of PtWFY-2 strain in Ca_3_(PO_4_)_2_ fermentation broth detected by LC-MS/MS; C The spectra of PtWFY-1 strain in Ca_3_(PO_4_)_2_ fermentation broth detected by HPLC; D The spectra of PtWFY-2 strain in Ca_3_(PO_4_)_2_ fermentation broth detected by HPLC. The spectra was recorded on day 5. PtWFY-1: PVK broth with PtWFY-1 inoculation; PtWFY-2: PVK broth with PtWFY-2 inoculation.


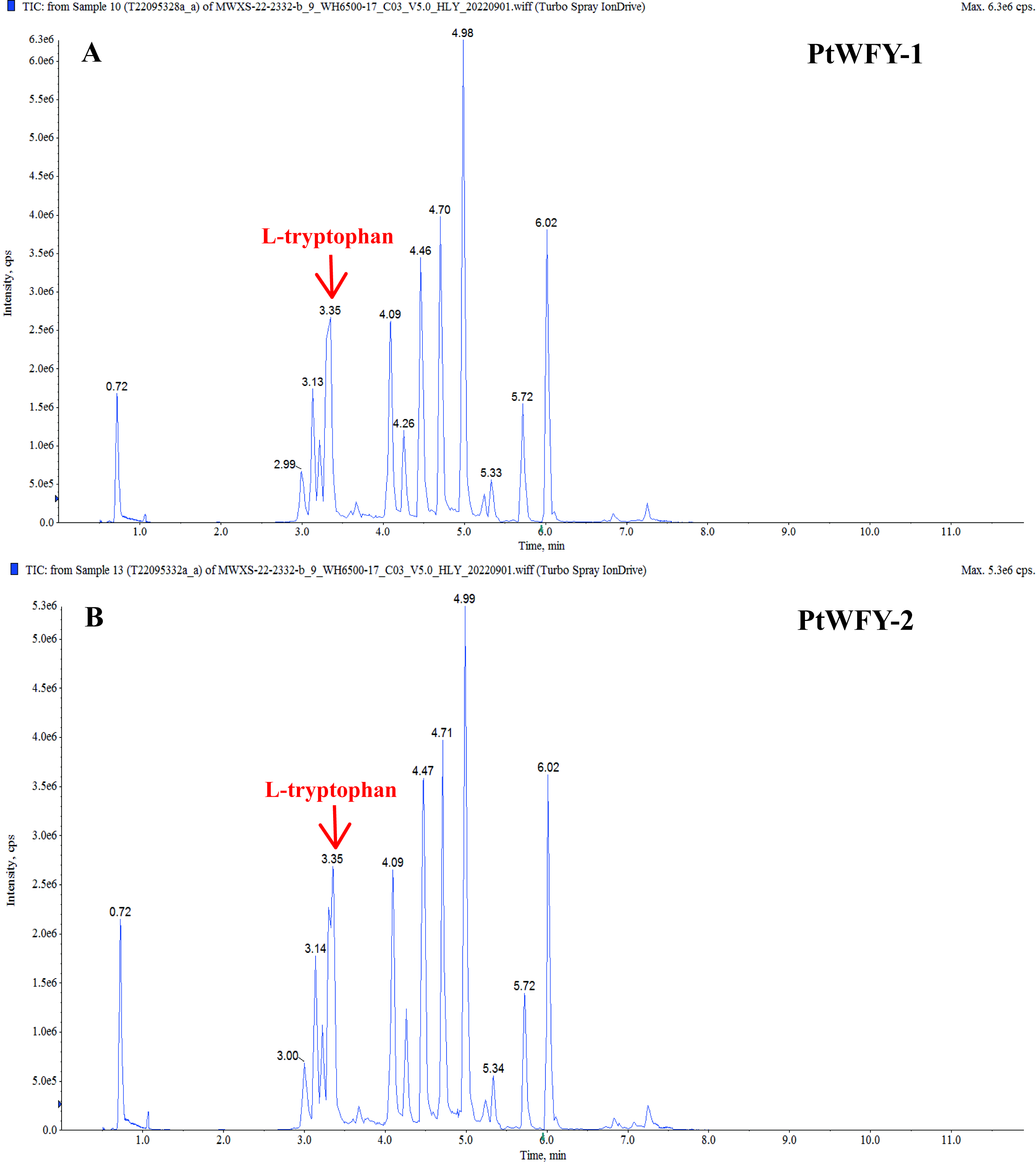


Fig. S4 The spectra of phytohormones secreted by *Pyrenochaetopsis tabarestanensis* WFY-1 (PtWFY-1) and WFY-2 (PtWFY-2). A The spectra of PtWFY-1 strain in PDB medium; B The spectra of PtWFY-2 strain in PDB medium. The spectra was recorded on day 5. PtWFY-1: PVK broth with PtWFY-1 inoculation; PtWFY-2: PVK broth with PtWFY-2 inoculation.

Table S1 The types and concentrations of organic acids secreted by PtWFY-1 and PtWFY-2 strains during in vitro solubilization of calcium phosphate in liquid medium (PVK).

| **Organic acid type** | **Organic acid concentration of Control** **(ng·ml^-1^)** | **Organic acid concentration of PtWFY-1** **(ng·ml^-1^)** | **Organic acid concentration of PtWFY-2** **(ng·ml^-1^)** |
| --- | --- | --- | --- |
| shikimic acid | 0.00 ± 0.00 | 0.00 ± 0.00 | 26.34 ± 7.18 |
| taurine | 0.00 ± 0.00 | 0.00 ± 0.00 | 2.80 ± 0.07 |
| neochlorogenic acid | 0.00 ± 0.00 | 0.00 ± 0.00 | 0.57 ± 0.12 |
| lactic acid | 0.00 ± 0.00 | 239.22 ± 26.70 | 0.00 ± 0.00 |
| maleic acid | 0.00 ± 0.00 | 14.40 ± 0.29 | 0.00 ± 0.00 |
| adipic acid | 0.00 ± 0.00 | 8.64 ± 0.54 | 0.00 ± 0.00 |
| oxoglutaric acid | 0.00 ± 0.00 | 1900.03 ± 101.73 | 2441.67 ± 121.40 |
| acetic acid | 0.00 ± 0.00 | 1478.47 ± 7.19 | 1519.18 ± 20.34 |
| pyruvic acid | 0.00 ± 0.00 | 685.90 ± 33.65 | 888.30 ± 86.05 |
| citric acid | 0.00 ± 0.00 | 579.11 ± 10.21 | 867.65 ± 9.62 |
| malonic acid | 0.00 ± 0.00 | 306.97 ± 2.16 | 289.56 ± 5.05 |
| formic acid | 0.00 ± 0.00 | 250.13 ± 4.81 | 328.86 ± 8.85 |
| succinic acid | 24.38 ± 0.68 | 205.17 ± 7.97 | 59.90 ± 5.50 |
| aconitate | 0.00 ± 0.00 | 175.60 ± 5.81 | 194.69 ± 4.41 |
| tartaric acid | 0.00 ± 0.00 | 166.49 ± 1.31 | 144.38 ± 15.10 |
| citraconic acid | 0.00 ± 0.00 | 132.73 ± 5.44 | 199.87 ± 22.87 |
| 5-hydroxymethyl-2-furoic acid | 0.00 ± 0.00 | 129.47 ± 0.78 | 19.42 ± 0.44 |
| oxalic acid | 0.00 ± 0.00 | 111.00 ± 3.88 | 121.25 ± 3.53 |
| L-malic acid | 0.00 ± 0.00 | 92.81 ± 1.81 | 154.17 ± 59.54 |
| 4-aminobutyric acid | 0.00 ± 0.00 | 30.96 ± 2.58 | 79.10 ± 6.13 |
| propionic acid | 0.00 ± 0.00 | 22.63 ± 0.76 | 25.35 ± 0.29 |
| fumaric acid | 0.00 ± 0.00 | 14.37 ± 1.62 | 20.69 ± 2.09 |
| pantothenic acid | 0.00 ± 0.00 | 10.49 ± 0.37 | 14.70 ± 1.38 |
| kynurenic acid | 0.00 ± 0.00 | 7.05 ± 0.06 | 19.94 ± 0.81 |
| aminobenzoic acid | 0.00 ± 0.00 | 4.13 ± 0.07 | 8.53 ± 0.48 |
| 4-hydroxybenzoic acid | 0.00 ± 0.00 | 3.59 ± 0.17 | 3.12 ± 0.09 |
| 3-phenyllactic acid | 0.00 ± 0.00 | 3.15 ± 0.06 | 3.57 ± 0.64 |
| hydroxyphenyllactic acid | 0.00 ± 0.00 | 2.03 ± 0.09 | 2.97 ± 0.19 |
| 3-hydroxymethylglutaric acid | 0.00 ± 0.00 | 1.58 ± 0.09 | 1.89 ± 0.28 |

The values represent the mean of three replicates ± Standard error.

Table S2 The types and concentrations of phytohormones secreted by PtWFY-1 and PtWFY-2 strains during in liquid medium (PDA).

| **Phytohormones type** | **Phytohormones concentration of Control** **(ng·ml^-1^)** | **Phytohormones concentration of PtWFY-1** **(ng·ml^-1^)** | **Phytohormones concentration of PtWFY-2** **(ng·ml^-1^)** |
| --- | --- | --- | --- |
| Gibberellin A20 | 0.37 ± 0.018 | 0.00 ± 0.00 | 0.38 ± 0.024 |
| Methyl jasmonate | 0.00 ± 0.00 | 0.00 ± 0.00 | 0.19 ± 0.0072 |
| 6-Benzyladenosine | 0.00 ± 0.00 | 0.00 ± 0.00 | 0.0014 ± 0.00006 |
| Dihydrozeatin-7-glucoside | 0.008 ± 0.00027 | 0.016 ±0.00067 | 0.00 ± 0.00 |
| N-(3-Indolylacetyl)-L-phenylalanine | 0.00 ± 0.00 | 0.0052 ± 0.00032 | 0.00 ± 0.00 |
| L-tryptophan | 2823.77 ± 77.84 | 3541.57 ± 17.13 | 3769.21 ± 20.80 |
| 1-Aminocyclopropanecarboxylic acid | 541.29 ± 2.96 | 559.54 ± 3.62 | 688.35 ± 2.40 |
| Indole-3-acetic acid | 1.94 ± 0.013 | 56.60 ± 1.06 | 37.00 ± 0.92 |
| 2-oxindole-3-acetic acid | 5.41 ± 0.18 | 7.19 ± 0.34 | 7.62 ± 0.25 |
| Indole-3-lactic acid | 1.44 ± 0.11 | 5.87 ± 0.050 | 4.17 ± 0.43 |
| N6-isopentenyladenosine | 0.68 ± 0.046 | 3.82 ± 0.14 | 1.91 ± 0.033 |
| Indole-3-carboxylic acid | 1.59 ± 0.093 | 2.58 ± 0.16 | 2.33 ± 0.021 |
| 3-Indole acetamide | 0.18 ± 0.011 | 0.95 ± 0.0067 | 0.28 ± 0.030 |
| cis-Zeatin riboside | 0.16 ± 0.0058 | 0.92 ± 0.011 | 0.63 ± 0.0063 |
| 2-Methylthio-N6-isopentenyladenosine | 0.42 ± 0.039 | 0.50 ± 0.036 | 0.43 ± 0.046 |
| Gibberellin A3 | 0.086 ± 0.0022 | 0.49 ± 0.041 | 0.59 ± 0.045 |
| Indole-3-acetyl glycine | 0.37 ± 0.013 | 0.34 ± 0.0058 | 0.51 ± 0.027 |
| meta-Topolin-9-glucoside | 0.29 ± 0.013 | 0.32 ± 0.015 | 0.18 ± 0.0073 |
| Dihydrozeatin ribonucleoside | 0.00 ± 0.00 | 0.24 ± 0.0096 | 0.39 ± 0.027 |
| N6-Isopentenyl-adenine-7-glucoside | 0.24 ± 0.0075 | 0.22 ± 0.0089 | 0.29 ± 0.031 |
| trans-Zeatin-O-glucoside | 0.19 ± 0.019 | 0.21 ± 0.012 | 0.24 ± 0.019 |
| 3-Indolepropionic acid | 0.27 ± 0.0064 | 0.16 ± 0.0089 | 0.32 ± 0.0089 |
| 3-Indoleacrylic acid | 0.10 ± 0.0032 | 0.13 ± 0.0042 | 0.23 ± 0.011 |
| trans-Zeatin | 0.041 ± 0.0039 | 0.12 ± 0.0032 | 0.14 ± 0.0049 |
| Indole-3-acetyl glutamic acid | 0.073 ± 0.0030 | 0.089 ± 0.0076 | 0.089 ± 0.0054 |
| Kinetin riboside | 0.072 ± 0.0043 | 0.081 ± 0.0014 | 0.084 ± 0.0038 |
| 2-Methylthio-cis-zeatin riboside | 0.023 ± 0.0023 | 0.067 ± 0.0073 | 0.079 ± 0.0047 |
| trans-Zeatin riboside | 0.031 ± 0.0030 | 0.049 ± 0.00069 | 0.056 ± 0.0052 |
| Dihydrozeatin | 0.044 ± 0.0030 | 0.048 ± 0.0032 | 0.044 ± 0.0037 |
| Jasmonic acid | 0.044 ± 0.0029 | 0.045 ± 0.018 | 0.025 ± 0.0024 |
| Gibberellin A9 | 0.060 ± 0.0033 | 0.043 ± 0.0047 | 0.073 ± 0.0033 |
| Methyl indole-3-acetate | 0.0065 ± 0.0003 | 0.041 ± 0.0018 | 0.10 ± 0.0088 |
| N6-Benzyladenine-9-glucoside | 0.00 ± 0.00 | 0.026 ± 0.0018 | 0.027 ± 0.0018 |
| cis-Zeatin | 0.015 ± 0.00118 | 0.016 ± 0.00015 | 0.069 ± 0.0022 |
| Indole-3-acetyl-L-valine methyl ester | 0.0021 ± 0.00007 | 0.0032 ± 0.00021 | 0.0044 ± 0.00028 |

The values represent the mean of three replicates ± Standard error.

Table S3 The standards used to measure the contents of organic acids secreted by PtWFY-1 and PtWFY-2 strains.

| **Index** | **RT** | **Equation** | **r** |
| --- | --- | --- | --- |
| 4-aminobutyric-acid | 0.72 | y = 26128.82898 x + 1504.95995 | 0.9996 |
| taurine | 0.74 | y = 3.56959e4 x + 1121.77561 | 0.9990 |
| L-malic-acid | 0.83 | y = 3920.65021 x + 5977.17160 | 0.9939 |
| shikimic-acid | 0.83 | y = 3312.83657 x + 2492.02388 | 0.9950 |
| tartaric-acid | 0.83 | y = 1232.40571 x - 7.54212e5 | 0.9905 |
| pyruvic-acid | 0.90 | y = 1199.16125 x + 5.62407e4 | 0.9942 |
| oxoglutaric-acid | 1.10 | y = 945.41826 x + 3.28726e4 | 0.9958 |
| lactic-acid | 1.10 | y = 960.27429 x + 1.65115e5 | 0.9922 |
| pyroglutamic-acid | 1.11 | y = 2.28509e5 x + 2.12303e5 | 0.9946 |
| maleic-acid | 1.12 | y = 12212.66881 x + 4.60208e4 | 0.9955 |
| cis-aconitic-acid | 1.21 | y = 4654.05575 x - 2.67228e6 | 0.9949 |
| fumaric-acid | 1.23 | y = 4916.62576 x + 18143.19889 | 0.9994 |
| succinic-acid | 1.30 | y = 16979.43850 x + 5.42528e4 | 0.9993 |
| 3-D-hydroxybutyric-acid | 1.34 | y = 7836.23413 x + 9.56490e4 | 0.9962 |
| trans-aconitic-acid | 1.35 | y = 4906.98879 x - 4.05320e5 | 0.9936 |
| 3-hydroxymethylglutaric-acid | 1.41 | y = 8020.32284 x + 605.59932 | 0.9990 |
| methylmalonic-acid | 1.45 | y = 11750.87544 x - 29105.13567 | 0.9955 |
| crtraconic-acid | 1.68 | y = 3839.74232 x + 808.93285 | 0.9952 |
| gallic-acid | 1.74 | y = 2.35570e5 x + 5187.95580 | 0.9992 |
| glutaric-acid | 1.88 | y = 2848.93985 x + 22476.43201 | 0.9982 |
| levulinic-acid | 1.91 | y = 1939.93621 x + 1.57115e5 | 0.9911 |
| kynurenine | 2.01 | y = 5950.56990 x + 21679.67261 | 0.9994 |
| 3-hydroxyisovaleric-acid | 2.05 | y = 3999.96945 x + 4372.17040 | 0.9998 |
| 5-hydroxymethyl-2-furoic-acid | 2.12 | y = 11132.76810 x + 4716.38040 | 0.9978 |
| pantothenic-acid | 2.28 | y = 13136.18690 x + 1682.27597 | 0.9983 |
| 2-methylsuccinic-acid | 2.29 | y = 13869.81524 x + 5.77495e4 | 0.9975 |
| ethylmalonic-acid | 2.37 | y = 6226.56885 x - 1.05950e5 | 0.9990 |
| 2-hydroxy-2-methylbutyric-acid | 2.45 | y = 7870.69025 x + 2253.26225 | 0.9989 |
| 4-hydroxyhippuric-acid | 2.49 | y = 7.66649e4 x + 1168.11568 | 0.9994 |
| neochlorogenic-acid | 2.58 | y = 7.90562e4 x - 881.32910 | 0.9956 |
| adipic-acid | 2.60 | y = 6079.86206 x + 23872.50586 | 0.9974 |
| 2-hydroxyisovaleric-acid | 2.66 | y = 19182.24229 x + 895.20578 | 0.9982 |
| 3-hydroxyhippuric-acid | 2.69 | y = 1.98072e5 x + 517.95840 | 0.9975 |
| 3,4-dihydroxyphenylacetic-acid | 2.73 | y = 167.33777 x + 12082.45253 | 0.9944 |
| hydroxyphenyllactic-acid | 2.74 | y = 22249.10952 x + 10385.13446 | 0.9960 |
| kynurenic-acid | 2.80 | y = 3.92815e5 x + 6520.89051 | 0.9972 |
| 3-Hydroxyphenyl-hydracrylic-acid | 2.82 | y = 28082.00050 x + 1707.42342 | 0.9981 |
| cryptochlorogenic-acid | 2.94 | y = 22156.77365 x - 6867.64836 | 0.9995 |
| 4-hydroxybenzoic-acid | 3.09 | y = 5.88130e4 x + 4.28764e4 | 0.9971 |
| 5-hydroxyindoleacetic-acid | 3.09 | y = 1.14610e5 x + 32.56256 | 0.9996 |
| 3-methyladipic-acid | 3.14 | y = 1.13279e5 x + 8.18571e4 | 0.9990 |
| 4-hydroxyphenylacetic-acid | 3.18 | y = 6342.87368 x + 1.08552e5 | 0.9959 |
| hippuric-acid | 3.23 | y = 21486.88832 x + 9223.79801 | 0.9980 |
| caffeic-acid | 3.24 | y = 8.03749e4 x + 20921.77276 | 0.9985 |
| homovanillic-acid | 3.36 | y = 1599.20816 x + 4.84278e4 | 0.9961 |
| 3-hydroxyphenylacetic-acid | 3.39 | y = 1000.24134 x + 4.09681e4 | 0.9976 |
| phenaceturic-acid | 3.50 | y = 1.10227e5 x + 1405.19954 | 0.9984 |
| 2-hydroxyphenylacetic-acid | 3.64 | y = 6.54327e4 x + 4.94533e4 | 0.9956 |
| suberic-acid | 3.69 | y = 3.28524e4 x + 3.81516e5 | 0.9990 |
| 4-coumaric-acid | 3.73 | y = 9.00399e4 x + 5071.81043 | 0.9980 |
| aminobenzoic-acid | 3.83 | y = 4.57934e5 x - 1750.89401 | 0.9980 |
| 3-phenyllactic-acid | 3.84 | y = 7454.59212 x + 2706.11965 | 0.9988 |
| ferulic-acid | 3.89 | y = 20971.53054 x + 182.29375 | 0.9971 |
| indolelactic-acid | 3.99 | y = 3.79753e4 x + 2351.79048 | 0.9985 |
| azelaic-acid | 4.20 | y = 5.79158e4 x + 1.57474e6 | 0.9995 |
| benzoic-acid | 4.35 | y = 1723.77100 x + 22876.35567 | 0.9985 |
| indole-3-acetic-acid | 4.44 | y = 1.45225e6 x + 7652.83921 | 0.9970 |
| salicylic-acid | 4.50 | y = 1.95808e5 x + 2.58577e5 | 0.9963 |
| sebacic-acid | 4.70 | y = 1.63370e5 x + 3.81865e6 | 0.9995 |
| 2-indolecarboxylic-acid | 4.91 | y = 3.44629e4 x + 22636.28657 | 0.9967 |
| benzenepropanoic-acid | 5.01 | y = 470.24102 x - 4462.46330 | 0.9957 |
| cinnamic-acid | 5.06 | y = 7.02783e4 x + 11516.64276 | 0.9970 |
| maslinic-acid | 8.21 | y = 34.37950 x + 8874.57208 | 0.9947 |
| carnosic-acid | 8.27 | y = 73.46953 x - 329.31751 | 0.9989 |
| oleanic-acid | 9.77 | y = 844.64235 x + 86.36901 | 0.9993 |
| oxalic acid | 4.29 | y = 4.8234x - 4.034 | 0.9905 |
| citric Acid | 13.57 | y = 48.556x - 42.097 | 0.9899 |
| tartaric Acid | 5.19 | y = 24.095x - 20.394 | 0.9897 |
| formic acid | 5.45 | y = 24.18x - 20.094 | 0.9899 |
| malonic acid | 7.44 | y = 24.126x - 20.478 | 0.9902 |
| acetic acid | 8.59 | y = 48.421x - 41.055 | 0.9898 |
| maleic acid | 12.28 | y = 0.2452x - 0.2064 | 0.9898 |
| aconitate | 17.81 | y = 47.79x - 39.936 | 0.9891 |
| propionic acid | 21.35 | y = 48.114x - 41.204 | 0.9893 |

Index: substance abbreviation; RT: retention time; Equation: Linear Equation; r: coefficient of correlation.

Table S4 The standards used to measure the contents of phytohormones secreted by PtWFY-1 and PtWFY-2 strains.

| **Index** | **Class** | **RT** | **Equation** | **r** |
| --- | --- | --- | --- | --- |
| ABA-glucosyl ester | ABA | 4.43 | y = 0.00151 x + 9.32183e^-5^ | 0.9915 |
| Abscisic acid | ABA | 5.26 | y = 0.11670 x + 5.34143e^-4^ | 0.9975 |
| L-tryptophan | Auxin | 3.31 | y = 3838.12685 x + 7789.66169 | 0.9994 |
| Tryptamine | Auxin | 3.60 | y = 6.55464e4 x + 11685.45074 | 0.9943 |
| 2-oxindole-3-acetic acid | Auxin | 4.19 | y = 0.00924 x + 2.82467e^-4^ | 0.9999 |
| Indole-3-acetyl-L-aspartic acid | Auxin | 4.25 | y = 0.02116 x + 3.35390e^-4^ | 0.9971 |
| 1-O-indol-3-ylacetylglucose | Auxin | 4.25 | y = 3.67456e-5 x + 1.48106e^-5^ | 0.9947 |
| 3-Indoleacetamide | Auxin | 4.29 | y = 0.02550 x + 7.39621e^-4^ | 0.9917 |
| Indoleacetyl glutamic acid | Auxin | 4.37 | y = 0.03746 x + 1.14163e^-4^ | 0.9976 |
| Indole-3-acetyl glycine | Auxin | 4.38 | y = 0.06853 x + 0.00349 | 0.9973 |
| Indole-3-lactic acid | Auxin | 4.60 | y = 0.00449 x + 0.00130 | 0.9986 |
| N-(3-Indolylacetyl)-L-alanine | Auxin | 4.68 | y = 0.10307 x + 0.00138 | 0.9990 |
| Indole-3-carboxylic Acid | Auxin | 4.73 | y = 0.01025 x + 2.88715e^-4^ | 0.9995 |
| Indole-3-carboxaldehyde | Auxin | 4.86 | y = 0.02292 x + 0.00172 | 0.9997 |
| Indole-3-acetic acid | Auxin | 4.99 | y = 0.01244 x + 2.18869e^-4^ | 0.9960 |
| 3-Indoleacrylic acid | Auxin | 5.21 | y = 0.01272 x + 2.40081e^-4^ | 0.9995 |
| N-(3-Indolylacetyl)-L-valine | Auxin | 5.36 | y = 0.17292 x + 0.00115 | 0.9961 |
| 3-Indolepropionic acid | Auxin | 5.41 | y = 0.03641 x - 7.07410e^-4^ | 0.9986 |
| Indole-3-acetyl-Lglutamic acid dimethyl ester | Auxin | 5.52 | y = 0.01351 x + 1.38597e^-4^ | 0.9978 |
| Indole-3-acetyl-L-tryptophan | Auxin | 5.70 | y = 0.06755 x + 5.56844e^-4^ | 0.1000 |
| N-(3-Indolylacetyl)-L-leucine | Auxin | 5.73 | y = 0.24042 x + 0.00625 | 0.9938 |
| 3-Indolebutyric acid | Auxin | 5.73 | y = 0.02094 x + 0.02575 | 0.9990 |
| 3-Indoleacetonitrile | Auxin | 5.75 | y = 0.00931 x + 7.41361e^-4^ | 0.9998 |
| N-(3-Indolylacetyl)-L-phenylalanine | Auxin | 5.81 | y = 0.12728 x + 0.00138 | 0.9988 |
| Methyl indole-3-acetate | Auxin | 6.04 | y = 0.04956 x + 3.23136e^-4^ | 0.9907 |
| Indole-3-acetyl-L-valine methyl ester | Auxin | 6.07 | y = 0.83385 x + 0.00643 | 0.9965 |
| Indole-3-acetyl-L-leucine methyl ester | Auxin | 6.41 | y = 0.72903 x + 0.00597 | 0.9901 |
| Indole-3-acetyl-L-phenylalanne methyle ester | Auxin | 6.46 | y = 0.63263 x + 0.00509 | 0.9929 |
| trans-Zeatin-O-glucoside | CK | 2.91 | y = 0.04821 x + 7.84255e^-4^ | 0.9992 |
| trans-Zeatin | CK | 3.04 | y = 0.16362 x + 0.00524 | 0.9992 |
| Dihydrozeatin | CK | 3.16 | y = 0.07569 x + 0.00255 | 0.9992 |
| Dihydrozeatin-7-glucoside | CK | 3.18 | y = 0.39834 x + 0.00183 | 0.9975 |
| cis-Zeatin | CK | 3.23 | y = 0.10013 x + 0.00186 | 0.9990 |
| cis-Zeatin-9-glucoside | CK | 3.28 | y = 0.39242 x + 4.21780e^-4^ | 0.9928 |
| Dihydrozeatin-O-glucoside riboside | CK | 3.48 | y = 0.21989 x + 6.96833e^-4^ | 0.9959 |
| cis-Zeatin-O-glucoside riboside | CK | 3.50 | y = 0.05675 x + 4.38462e^-5^ | 0.9988 |
| para-Topolin | CK | 3.54 | y = 0.07163 x + 9.45010e^-4^ | 0.9988 |
| 4-[[(9-beta-D-Glucopyranosyl-9H-purin-6-yl)amino]methyl]phenol | CK | 3.55 | y = 0.17326 x + 3.87279e^-4^ | 0.9937 |
| trans-Zeatin riboside | CK | 3.58 | y = 0.33210 x + 0.00101 | 0.9961 |
| Dihydrozeatin ribonucleoside | CK | 3.59 | y = 0.18714 x + 7.25188e^-4^ | 0.9924 |
| cis-Zeatin riboside | CK | 3.67 | y = 0.19371 x + 0.00131 | 0.9952 |
| N6-Isopentenyl-adenine-7-glucoside | CK | 3.70 | y = 0.41890 x + 0.00138 | 0.9998 |
| meta-Topolin-9-glucoside | CK | 3.71 | y = 0.18541 x + 0.00116 | 0.9941 |
| meta-Topolin | CK | 3.71 | y = 0.10091 x + 0.00137 | 0.9992 |
| Kinetin | CK | 3.78 | y = 0.07683 x + 9.12811e^-4^ | 0.9996 |
| Kinetin-9-glucoside | CK | 3.79 | y = 0.25268 x + 0.00141 | 0.9933 |
| N6-Benzyladenine-7-glucoside | CK | 3.80 | y = 0.56164 x + 0.00418 | 0.9998 |
| para-Topolin riboside | CK | 3.93 | y = 0.19060 x + 0.00142 | 0.9993 |
| N6-Isopentenyl-adenine-9-glucoside | CK | 4.07 | y = 0.28146 x + 4.79044e^-4^ | 0.9906 |
| meta-Topolin riboside | CK | 4.08 | y = 0.32897 x + 7.59431e^-4^ | 0.9901 |
| ortho-Topolin-9-glucoside | CK | 4.09 | y = 0.14433 x + 3.80111e^-4^ | 0.9959 |
| N6-isopentenyladenine | CK | 4.12 | y = 0.09525 x + 0.00121 | 0.9917 |
| ortho-Topolin | CK | 4.13 | y = 0.17088 x + 6.50455e^-4^ | 0.9926 |
| Kinetin riboside | CK | 4.20 | y = 0.31455 x + 3.64184e^-4^ | 0.9906 |
| N6-Benzyladenine -9-glucoside | CK | 4.24 | y = 0.40849 x + 0.00252 | 0.9906 |
| 6-Benzyladenine | CK | 4.29 | y = 0.17423 x + 0.00223 | 0.9911 |
| 2-Chloro-trans-zeatin | CK | 4.40 | y = 0.07569 x + 5.69721e^-4^ | 0.9923 |
| ortho-Topolin riboside | CK | 4.45 | y = 0.26717 x + 0.00247 | 0.9985 |
| N6-isopentenyladenosine | CK | 4.49 | y = 0.10840 x + 8.51558e^-4^ | 0.9916 |
| 2-methylthio-cis-zeatin | CK | 4.50 | y = 0.04263 x + 0.00131 | 0.9993 |
| 2-Methylthio-cis-zeatin riboside | CK | 4.56 | y = 0.19138 x + 0.00240 | 0.9965 |
| 6-Benzyladenosine | CK | 4.63 | y = 0.61572 x + 0.00382 | 0.9963 |
| 2-Methylthio-N6-isopentenyladenosine | CK | 5.74 | y = 0.04382 x + 4.85798e^-4^ | 0.9994 |
| 2-Methylthio-N6-isopentenyladenine | CK | 5.84 | y = 0.03534 x + 0.00110 | 0.9994 |
| 1-Aminocyclopropanecarboxylic acid | ETH | 0.72 | y = 13977.79709 x + 14178.28663 | 0.9993 |
| Gibberellin A3 | GA | 4.41 | y = 0.05540 x + 0.02051 | 0.9999 |
| Gibberellin A1 | GA | 4.45 | y = 0.01762 x + 0.01275 | 0.9966 |
| Gibberellin A19 | GA | 5.13 | y = 0.04180 x + 0.00196 | 0.9940 |
| Gibberellin A20 | GA | 5.31 | y = 0.08253 x + 0.00392 | 0.9981 |
| Gibberellin A53 | GA | 5.71 | y = 0.07635 x + 0.04869 | 0.9961 |
| Gibberellin A7 | GA | 6.06 | y = 0.35547 x + 0.00473 | 0.9936 |
| Gibberellin A4 | GA | 6.13 | y = 0.04612 x + 0.00219 | 0.9984 |
| Gibberellin A24 | GA | 6.31 | y = 0.08504 x + 8.10155e^-4^ | 0.9907 |
| Gibberellin A15 | GA | 6.84 | y = 0.06608 x + 0.00145 | 0.9999 |
| Gibberellin A9 | GA | 6.85 | y = 0.10601 x + 0.00296 | 0.9981 |
| Jasmonic Acid | JA | 5.76 | y = 0.08592 x + 0.00322 | 0.9984 |
| N-[(-)-Jasmonoyl]-(L)-valine | JA | 5.97 | y = 0.96511 x + 0.00382 | 0.9917 |
| Dihydrojasmonic acid | JA | 6.13 | y = 0.11283 x + 0.00364 | 0.9950 |
| Jasmonoyl-L-isoleucine | JA | 6.30 | y = 0.28397 x + 0.00186 | 0.9914 |
| N-[(-)-Jasmonoyl]-(l)-phenalanine | JA | 6.35 | y = 0.71968 x + 0.00763 | 0.9901 |
| 3-oxo-2-(2-(Z)-Pentenyl) cyclopentane-1-butyric acid | JA | 6.36 | y = 0.06731 x + 0.04843 | 0.9936 |
| Methyl jasmonate | JA | 6.88 | y = 0.60087 x + 0.01723 | 0.9996 |
| 3-oxo-2-(2-(Z)-Pentenyl)cyclopentane-1-hexanoic acid | JA | 6.98 | y = 0.04056 x + 0.01111 | 0.9976 |
| cis(+)-12-Oxophytodienoic acid | JA | 7.23 | y = 1.88724 x + 0.05384 | 0.9903 |
| Salicylic acid 2-O-β-Glucoside | SA | 3.55 | y = 0.03450 x + 0.00254 | 0.9950 |
| Salicylic Acid | SA | 5.03 | y = 0.10099 x + 0.01138 | 0.9999 |
| （±）Strigol | SL | 6.35 | y = 589.53527 x + 178.11343 | 0.9965 |
| 5-Deoxystrigol | SL | 7.46 | y = 18617.98977 x + 2790.18589 | 0.9974 |

Index: substance abbreviation; RT: retention time; Equation: Linear Equation; r: coefficient of correlation.
